# Structural and mechanistic insights into ATRX-dependent and -independent functions of the histone chaperone DAXX

**DOI:** 10.1038/s41467-017-01206-y

**Published:** 2017-10-30

**Authors:** Dominik Hoelper, Hongda Huang, Aayushi Y. Jain, Dinshaw J. Patel, Peter W. Lewis

**Affiliations:** 1 Department of Biomolecular Chemistry, School of Medicine and Public Health, University of Wisconsin, Madison, WI 53706 USA; 2 Wisconsin Institute for Discovery, University of Wisconsin, Madison, WI 53715 USA; 3 Structural Biology Program, Memorial Sloan-Kettering Cancer Center, New York, NY 10065 USA; 4 Present Address: Department of Biology, South University of Science and Technology, Shenzhen, Guangdong 518055 China

## Abstract

The ATRX–DAXX histone chaperone complex incorporates the histone variant H3.3 at heterochromatic regions in a replication-independent manner. Here, we present a high-resolution x-ray crystal structure of an interaction surface between ATRX and DAXX. We use single amino acid substitutions in DAXX that abrogate formation of the complex to explore ATRX-dependent and ATRX-independent functions of DAXX. We find that the repression of specific murine endogenous retroviruses is dependent on DAXX, but not on ATRX. In support, we reveal the existence of two biochemically distinct DAXX-containing complexes: the ATRX–DAXX complex involved in gene repression and telomere chromatin structure, and a DAXX–SETDB1–KAP1–HDAC1 complex that represses endogenous retroviruses independently of ATRX and H3.3 incorporation into chromatin. We find that histone H3.3 stabilizes DAXX protein levels and can affect DAXX-regulated gene expression without incorporation into nucleosomes. Our study demonstrates a nucleosome-independent function for the H3.3 histone variant.

## Abstract (web summary)

The ATRX-DAXX histone chaperone complex incorporates H3.3 in heterochromatin in a replication-independent manner. Here, the authors present a high-resolution x-ray crystal structure of an interaction surface between ATRX and DAXX, and characterize ATRX-dependent and-independent functions of DAXX.

## Introduction

In addition to canonical histone proteins, multicellular organisms possess several histone variants that function in diverse nuclear processes. Histone variants, such as histone H3.3, are enriched at select genomic regions by specific deposition machineries and contain variant-specific residues and post-translational modifications^1, 2^. Initial characterization of histone H3.3 found it enriched at sites of active transcription via deposition by the histone regulator A (HIRA) complex^3, 4^. Later studies revealed that histone H3.3 is also deposited in heterochromatic regions, including telomeres and pericentric heterochromatin^5–8^. Incorporation of H3.3 at these silent genomic loci depended on a histone chaperone complex comprised of the death-domain-associated protein (DAXX) and the alpha-thalassemia/mental retardation X-linked syndrome protein (ATRX)^6, 8, 9^. In structural and biochemical studies, we and others previously demonstrated that DAXX directly interacts with an H3.3–H4 heterodimer via its highly conserved histone-binding domain^9–11^. ATRX is not required for interaction of DAXX with H3.3–H4, but instead serves to target DAXX-dependent H3.3 deposition at H3K9me3-enriched chromatin and telomeres through a conserved ATRX–DNMT3–DNMT3L (ADD) domain^9, 12^. Prior to its identification as a histone H3.3 chaperone, earlier investigations identified DAXX as a transcriptional co-repressor that interacts with histone deacetylases (HDAC) and DNA methyltransferases^13–16^. Very recent studies have implicated DAXX and ATRX in chromatin-based repression of long terminal repeat (LTR)-containing retrotransposons in an H3.3-dependent manner and through interaction with the SETDB1 and KAP1 co-repressor proteins^17, 18^. SETDB1 is an H3K9-directed methyltransferase that is targeted to endogenous retroviruses (ERVs) through interaction with KAP1 and KRAB-zinc finger proteins for ERV repression in mESCs^19, 20^. ERVs can promote genome instability through their insertion into protein coding regions or by inducing aberrant transcription of neighboring genes^21^. Furthermore, recent studies have linked the abnormal expression of ERVs and other repeat elements with the initiation of tumorigenesis^22–24^.

Inactivating mutations of *ATRX* and *DAXX* are frequently found in pancreatic neuroendocrine tumors, myelodysplastic syndrome, relapsed acute myeloid leukemia, neuroblastoma, and glioblastoma^25–27^. Tumors with *ATRX* or *DAXX* mutations exhibit an alternative lengthening of telomeres phenotype^28, 29^, supporting previous mechanistic studies that revealed essential functions of ATRX and DAXX in the maintenance of chromatin structure at telomeres^5, 6, 9^. Interestingly, most missense mutations in DAXX found in human tumors map to either the highly conserved histone-binding domain or the N-terminal four-helix bundle domain (4HB), indicating the importance of these domains in the putative tumor suppressor activity of DAXX. Previous studies found that the 4HB domain in DAXX interacts with several nuclear proteins, including the tumor suppressor RASSF1C, the E3 ubiquitin-ligase MDM2, as well as ATRX^30–32^. A DAXX-binding region was previously mapped to 138 residues in ATRX^31^. While the structural basis for the RASSF1C interaction with the DAXX 4HB has been described^33^, the molecular details for the ATRX–DAXX interaction are unknown.

Here, we present a crystal structure of the DAXX 4HB and a conserved helical DAXX-binding motif (DBM) in ATRX. Disruption of this ATRX–DAXX interaction surface through single amino acid substitutions has functional consequences in vivo. Specifically, loss of the ATRX–DAXX complex recapitulates select gene expression changes caused by genetic depletion of *ATRX* or *DAXX* genes; however, DAXX-dependent transcriptional repression of retrotransposons remains unaffected. Furthermore, we find that DAXX resides in at least two biochemically and functionally distinct complexes, and we provide details into the ATRX-independent interaction of DAXX with the SETDB1–KAP1 co-repressor proteins. Additionally, we demonstrate that the previously reported role of histone H3.3 in retrotransposon repression occurs independent of its deposition into nucleosomes. Instead, we show that histone H3.3 is functionally linked to retrotransposon repression through its stabilization of DAXX protein levels by direct interaction. These studies provide mechanistic and functional insights into biochemically distinct DAXX-containing chromatin modifying complexes in mammalian cells.

## Results

### Structural basis underlying the interaction of ATRX and DAXX

**Fig. 1:**
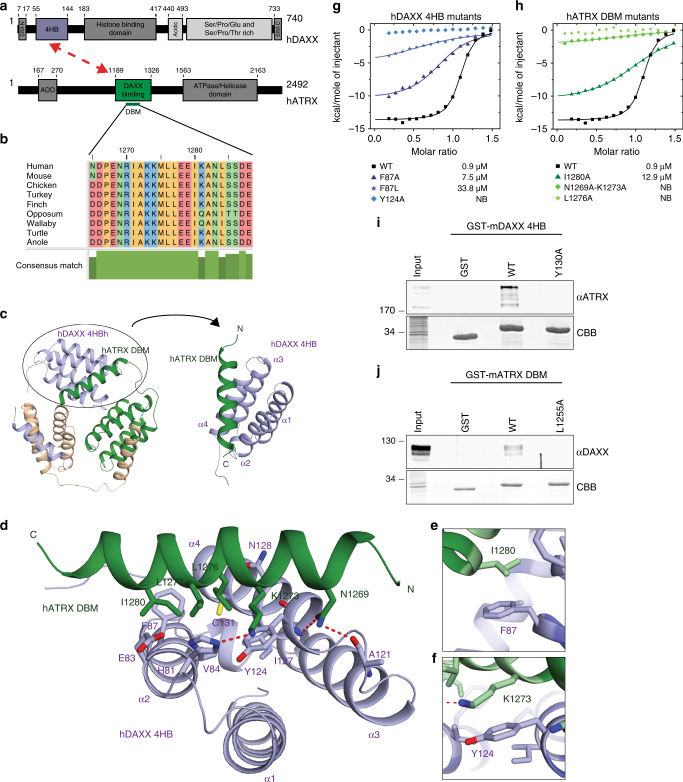
Structure and analyses of an ATRX–DAXX interaction surface. **a** Domain structures of human DAXX (hDAXX) and hATRX. ATRX interacts with the N-terminal four-helix bundle (4HB) in DAXX via a central region^31^. **b** Alignment showing residues of the highly conserved DAXX-interacting segment of hATRX (DAXX-binding motif, DBM) within the indicated vertebrate species. **c** Crystal structure showing the intermolecular interactions within the artificial trimer of the hDAXX 4HB–hATRX DBM fusion protein with each monomer highlighted in a different color (left). Ribbon view showing the α2 and α4 helices of hDAXX 4HB bound to the hATRX DBM (right). **d**–**f** Details of the interactions between hDAXX 4HB and hATRX DBM. An overview is shown in **d**. **e** and **f** Show zoomed-in views on the contacts between F87 in hDAXX 4HB and I1280 in hATRX DBM, as well as between Y124 in hDAXX 4HB and K1273 in hATRX DBM, respectively. **g**, **h** ITC results validating the observed interactions of DAXX 4HB and ATRX DBM in **d**. Dissociation constants (*K*_d_) of binding are shown. **i** Pulldown of full-length ATRX from murine embryonic fibroblast (MEF) nuclear extract using GST-tagged mouse DAXX (mDAXX) 4HB (WT and Y130A mutant) as bait. Immunoblot for ATRX and Coomassie stain of GST-tagged proteins in the eluate. **j** Pulldown of full-length DAXX from MEF nuclear extract using GST-tagged mATRX DBM (WT and L1255A) as bait. Immunoblot for DAXX and Coomassie stain of GST-tagged proteins in the elution are shown

Despite its noted importance in animal development and diseased states, little is known about how the ATRX–DAXX complex forms or about the interaction surfaces that mediate complex assembly. A previous study identified the 4HB domain of DAXX and a 138-residue fragment of ATRX as putative contact surfaces between the two proteins (Fig. 1a)^31^. We identified a short peptide of ATRX that interacted with the DAXX 4HB domain using different truncated GST-tagged versions of ATRX (Supplementary Fig. 1b, c). In order to gain mechanistic insight into the formation of the complex, we performed structural analyses of the interaction between human DAXX and ATRX. Our studies reveal that the DAXX 4HB forms extensive interactions with a short fragment, named DBM, of ATRX (Fig. 1a). The region of ATRX that interacts with the DAXX 4HB is highly conserved among vertebrate species (Fig. 1b; Supplementary Fig. 1a). Binding between the DAXX 4HB and ATRX DBM was stable at 1 M NaCl, a finding indicative of a hydrophobic interaction surface between the two proteins (Supplementary Fig. 1d). In order to facilitate crystallization, we generated a DAXX 4HB–ATRX DBM fusion protein, with the fusion cassette crystallizing as an intermolecular artificial trimer (Fig. 1c, left; Supplementary Fig. 1e). The α2 and α4 helices of DAXX 4HB form a surface to capture the ATRX DBM, which also adopts an α-helical conformation (Fig. 1c, right). The hydrocarbon side chain of ATRX I1280 packs against the aromatic ring of DAXX F87 (Fig. 1d, e). Also, the side chain of ATRX N1269 forms putative hydrogen bonds with the backbone atoms of DAXX A121 and Y124 (Fig. 1d), while the side chain of ATRX K1273 packs against the aromatic ring of DAXX Y124 (Fig. 1f; Supplementary Fig. 1f). Moreover, the side chains of ATRX L1276, L1277, and I1280 insert into a relatively hydrophobic groove between the α2 and α4 helices of DAXX 4HB, formed by DAXX H81, E83, V84, and F87 residues from the α2 helix and DAXX Y124, I127, N128, and C131 residues from the α4 helix (Fig. 1d). We used Isothermal Titration Calorimetry (ITC) to validate the observed interactions from the crystal structure (Fig. 1g, h). The measured *K*_d_ for the DAXX 4HB and ATRX DBM is 0.9 μM, with the DAXX Y124A mutation totally disrupting the interaction (Fig. 1g). We found that a tumor mutation (F87L) in DAXX reduces the binding affinity with the ATRX DBM to 33.8 μM (about 38-fold reduction) (Fig. 1g; Supplementary Fig. 6a). Conversely, the ATRX single mutant L1276A and dual mutant N1269A–K1273A also totally disrupt the interaction, and the ATRX I1280A mutant shows weakened binding by about 14-fold (Fig. 1h). The ITC results support the intermolecular interactions observed in the crystal structure.

To address whether the DAXX 4HB was sufficient to interact with full-length ATRX, we performed pulldown assays with GST-tagged DAXX 4HB in murine embryonic fibroblast (MEF) nuclear extract. Wild-type (WT) DAXX 4HB can interact with full-length ATRX in MEF nuclear extract, while the Y130A mutation in murine DAXX (analogous to Y124A in human DAXX) as well as mutations at F93 (analogous to F87 in human DAXX) fail to interact (Fig. 1i; Supplementary Fig. 2). Moreover, the ATRX DBM was also sufficient to pulldown full-length DAXX from nuclear extract (Fig. 1j). Thus, our structural and biochemical analyses have identified the molecular basis underlying an interaction within the ATRX–DAXX complex.

### Transcriptional repression and H3.3 deposition by ATRX–DAXX

**Fig. 2:**
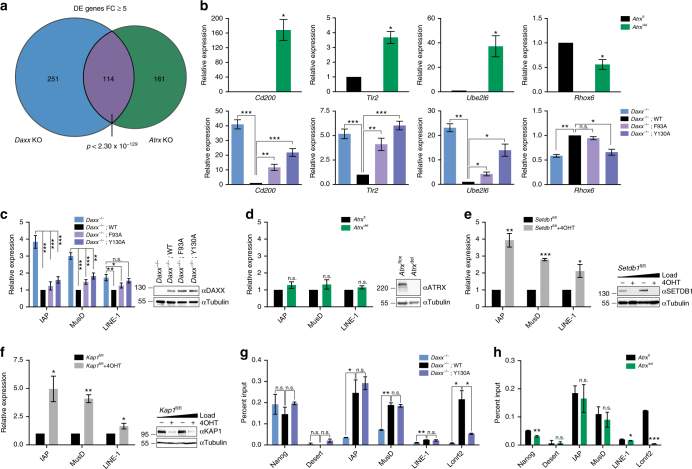
Analysis of DAXX-dependent and ATRX-dependent gene and retroelement expression in mESCs. **a** Venn diagram showing the overlap of genes differentially expressed in *Daxx* knockout (KO) and *Atrx* KO mESCs derived from publicly available RNA-Seq data (GEO accession no.: GSE73881). The *p*-value was determined by using hypergeometric distribution. **b** Validation of selected overlapping differentially expressed genes by RT-qPCR in *Atrx* ^fl^ and *Atrx* ^del^ mESCs, as well as *Daxx* ^−/−^ mESCs, rescued with different *Daxx* transgenes, as indicated. Data are presented as relative expression and were normalized to *Actb*. Shown are results from at least three independent experiments (error bars depict S.E.M.). **c**–**f** Expression of IAP, MusD and LINE-1 elements determined by RT-qPCR in **c** *Daxx* ^−/−^ mESCs, rescued with *Daxx* transgenes (WT, F93A, Y130A), in **d** *Atrx* ^fl^ and *Atrx* ^del^ mESCs, in **e** *Setdb1* ^fl/fl^ and 4-hydroxy tamoxifen-(4OHT)treated *Setdb1* ^fl/fl^ mESCs, and in **f** *Kap1* ^fl/fl^ and *Kap1* ^fl/fl^ +4OHT mESCs. Data are presented as relative expression and were normalized to *Actb* or *Gapdh*. Shown are results from at least three independent experiments (error bars depict S.E.M.). Immunoblots to confirm transgene expression or absence of target protein are shown adjacent to each panel. **g**, **h** Anti-HA ChIP-qPCR for indicated genomic regions in mESCs with stable H3.3-HA knock-in. **g** Shows *Daxx* ^−/−^ mESCs, rescued with *Daxx* transgenes (WT, Y130A); **h** shows *Atrx* ^fl^ and *Atrx* ^del^ mESCs. Data are presented as per cent input (error bars depict S.E.M.). Shown are the results of two independent experiments. Asterisks in **b** through **h** denote significance by Student’s *t*-test: *p < 0.05, **p < 0.01, ***p < 0.001, n.s. not significant

The ATRX–DAXX complex is implicated in transcription regulation through deposition of histone H3.3^34, 35^. We analyzed RNA-sequencing (RNA-Seq) data from *Daxx* knockout (KO) and *Atrx* KO murine embryonic stem cells (mESCs) to assess how disruption of the ATRX–DAXX complex affects gene expression. First, we found a significant overlap between genes differentially expressed (DE) in *Daxx* and *Atrx* KO mESCs (Fig. 2a). Our analysis suggests that interaction between ATRX and DAXX is required for regulation of genes in this data set (Fig. 2b). *Daxx* ^−/−^ cells rescued with *Daxx* transgenes containing F93A or Y130A resemble the expression pattern in *Daxx* ^−/−^ mESCs. These results indicate that disruption of the ATRX–DAXX complex by single residue substitutions phenocopy genetic depletion of either *Atrx* or *Daxx* genes with respect to ATRX–DAXX-regulated genes.

A recent report implicated the deposition of histone H3.3 by the ATRX–DAXX complex in the transcriptional repression of ERVs, particularly intracisternal A particle (IAP) elements^18^. This study proposed that ATRX–DAXX interacts with SETDB1–KAP1 co-repressor proteins to facilitate targeted H3.3 deposition and trimethylation of lysine 9 on histone H3 (H3K9me3) at ERVs. We assessed the expression of ERV elements IAP and *Mus musculus* type D (MusD), as well as the non-LTR retrotransposon long interspersed nuclear element 1 (LINE-1) in *Daxx* ^−/−^ mESCs rescued with different *Daxx* transgenes. We found that *Daxx* ^−/−^ mESCs show a 4-fold and 3-fold upregulation of IAP and MusD elements, respectively, compared to mESCs rescued with a WT *Daxx* transgene (Fig. 2c), while the expression of LINE-1 elements was not strongly affected by loss of *Daxx* (less than twofold upregulation). Interestingly, ATRX binding-deficient *Daxx* transgenes (F93A and Y130A) repressed expression of IAP and MusD elements to nearly the same extent as WT DAXX (Fig. 2c). These data suggest that DAXX-mediated repression of ERV elements occurs independently of interaction with ATRX. Indeed, we found that ERV repression remained unchanged in cells with reduced ATRX levels (by shRNA-mediated knockdown) or loss of ATRX by *Atrx* gene depletion (using CRE-mediated recombination) (Fig. 2d; Supplementary Fig. 3a). Only the loss of *Daxx* by genetic depletion exhibited ERV expression comparable to the loss of *Setdb1* or *Kap1* (Fig. 2e, f). We sought to determine if KAP1 and DAXX repress ERVs cooperatively or independently of each other. To this end, we assessed ERV expression upon siRNA-mediated knockdown of *Kap1* in the presence or absence of DAXX and ATRX. Knockdown of *Kap1* caused significant de-repression of ERVs in *Daxx* ^−/−^ mESCs rescued with a WT *Daxx* transgene, as well as in both *Atrx* ^fl^ and *Atrx* ^del^ mESCs (Supplementary Fig. 3c–e). However, *Kap1* knockdown did not result in additional upregulation of ERVs in *Daxx* ^−/−^ mESCs, indicating that ERVs are already maximally de-repressed in cells lacking DAXX (Supplementary Fig. 3b). These experiments confirm that DAXX—but not ATRX—functionally interacts with SETDB1–KAP1 co-repressor proteins in the process of ERV repression.

The ATRX-independent repression of ERVs by DAXX led us to investigate the role of H3.3 deposition in repression of ERVs. A previous study found that H3.3 was enriched at LTRs of IAPs in an ATRX-dependent and DAXX-dependent manner^18^. We performed chromatin immunoprecipitation (ChIP) on *Daxx* ^−/−^ mESCs in which the endogenous locus of *H3f3b* (one of two H3.3-encoding genes) had been engineered with a C-terminal hemagglutinin-(HA)epitope tag^9^. ChIP analysis showed that histone H3.3 was enriched at IAP and MusD elements in a DAXX-dependent manner (Fig. 2g), but independently of ATRX (Fig. 2h). Interestingly, the ATRX binding-deficient *Daxx* transgene Y130A did not exhibit significant differences in H3.3 enrichment at IAP and MusD elements as compared to WT *Daxx*. As a control, we assessed H3.3 deposition at a non-ERV short tandem repeat within an intron of the *Lonrf2* gene. H3.3 enrichment at this locus was lost upon genetic depletion of *Atrx* or *Daxx*, and could not be rescued by the Y130A *Daxx* transgene (Fig. 2g, h). These data show that H3.3 deposition at the LTRs of ERVs occurs independent of ATRX, while deposition of H3.3 at other repeats (such as short tandem repeats and telomeres) are dependent on ATRX.

### Existence of two biochemically distinct DAXX complexes

**Fig. 3:**
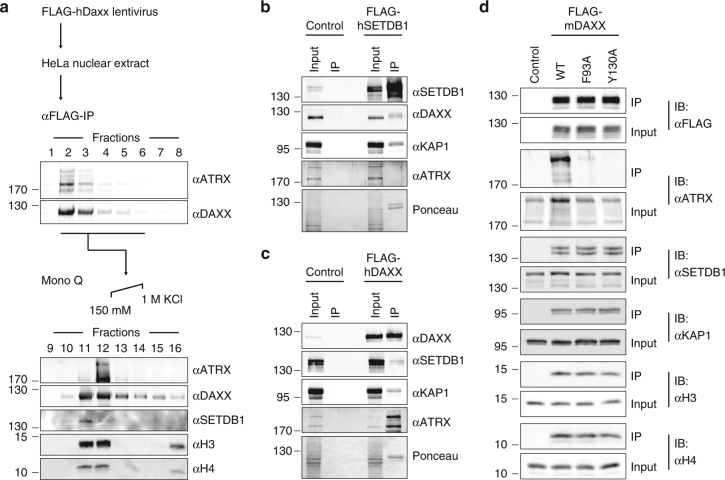
DAXX associates with SETDB1–KAP1 co-repressor proteins independently of ATRX. **a** HeLa nuclear extract containing FLAG-hDAXX was subjected to anti-FLAG IP. Eluates were analyzed by immunoblot for DAXX and ATRX (top). Elutions 2–6 were pooled and subjected to anion exchange chromatography (Mono Q) with a gradient of 150 mM to 1 M KCl (bottom). Shown are immunoblots of Mono Q fractions with antibodies for ATRX, DAXX, SETDB1, and histones H3 and H4. **b** 293T cells transfected with no construct (control) or FLAG-hSETDB1 were subjected to anti-FLAG IP. **c** 293T cells transfected with no construct (control) or FLAG-hDAXX were subjected to anti-FLAG IP. Inputs and eluates in **b** and **c** were analyzed for SETDB1, DAXX, ATRX, and KAP1 by immunoblot. Ponceau stains show the exogenous FLAG-tagged proteins present in the elution samples. **d** Immunoblots of input and eluates from FLAG-tagged mDAXX (WT, F93A, and Y130A) and control (no FLAG-tagged transgene) immunoprecipitation from MEF nuclear extract

Our data indicate that ERV repression in mESCs is dependent on DAXX, but not on the ATRX–DAXX complex. Previous fractionation of nuclear extracts found that a substantial amount of DAXX–H3.3 exists in complexes that are biochemically separable from ATRX–DAXX–H3.3^9^. We used a biochemical approach to investigate and dissect the nexus of DAXX–H3.3-containing complexes. To this end, we generated HeLa S3 cells stably expressing FLAG-tagged human DAXX and immunoprecipitated FLAG-DAXX and associated proteins from a nuclear extract (Fig. 3a, top). Interestingly, FLAG-DAXX eluate contained histone methyltransferase activity that effectively methylated unmodified H3^aa1–20^ peptide in vitro (Supplementary Fig. 4a, b). In an effort to identify the histone methyltransferase, we fractionated the FLAG-DAXX co-immunoprecipitated proteins by means of anion exchange chromatography (Mono Q). The H3K9-directed histone methyltransferase SETDB1 co-immunoprecipitated with DAXX from HeLa cell and mESC extract (Fig. 3a; Supplementary Fig. 4c). Immunoblot analysis revealed that the column fraction containing DAXX and SETDB1 had relatively little amounts of ATRX; conversely, the fraction containing the majority of ATRX had only small amounts of SETDB1 (Fig. 3a, bottom). The presence of SETDB1 in the FLAG-DAXX eluate is consistent with the previously noted co-immunoprecipitation of KAP1 with DAXX^18^. Furthermore, our results support the likelihood of at least two biochemically distinct DAXX-containing complexes in the FLAG-DAXX eluate.

We found that epitope-tagged SETDB1 effectively co-immunoprecipitated DAXX from 293T cell extract (Fig. 3b). Additionally, DAXX, but not SETDB1, co-immunoprecipitated ATRX in 293T cells (Fig. 3b, c). These data support a model of mutually exclusive interactions between DAXX and ATRX or SETDB1–KAP1. To directly address if ATRX interaction with DAXX is mutually exclusive to SETDB1–KAP1 binding, we performed immunoprecipitation of murine DAXX (WT, F93A, and Y130A) from stably transduced MEFs. Neither of the ATRX binding-deficient DAXX mutants affected interaction with SETDB1–KAP1 or with histones H3.3 and H4 (Fig. 3d). These data support the conclusion that interaction between DAXX and SETDB1–KAP1 occurs independent of ATRX. In support, expression patterns of ATRX–DAXX-regulated genes remained unchanged upon loss of SETDB1 or KAP1 (Supplementary Fig. 5a–c), indicating that SETDB1 and KAP1 do not participate in ATRX–DAXX-dependent gene regulation. We observed that steady-state levels of ATRX are influenced by the presence of and interaction with DAXX in MEFs. ATRX levels increased upon expression of WT DAXX, while less ATRX was detected upon expression of ATRX binding-deficient DAXX mutants F93A, F93L, or Y130A (Fig. 3d; Supplementary Fig. 6a). Importantly, we did not observe DAXX-dependent changes in *Atrx* mRNA levels (Supplementary Fig. 6b), suggesting that interaction between ATRX and DAXX likely stabilizes the ATRX protein. Conversely, DAXX protein levels are not dependent on the presence of ATRX (Supplementary Fig. 3a)^9^.

### H3.3 impacts ERV repression by stabilizing the DAXX protein

**Fig. 4:**
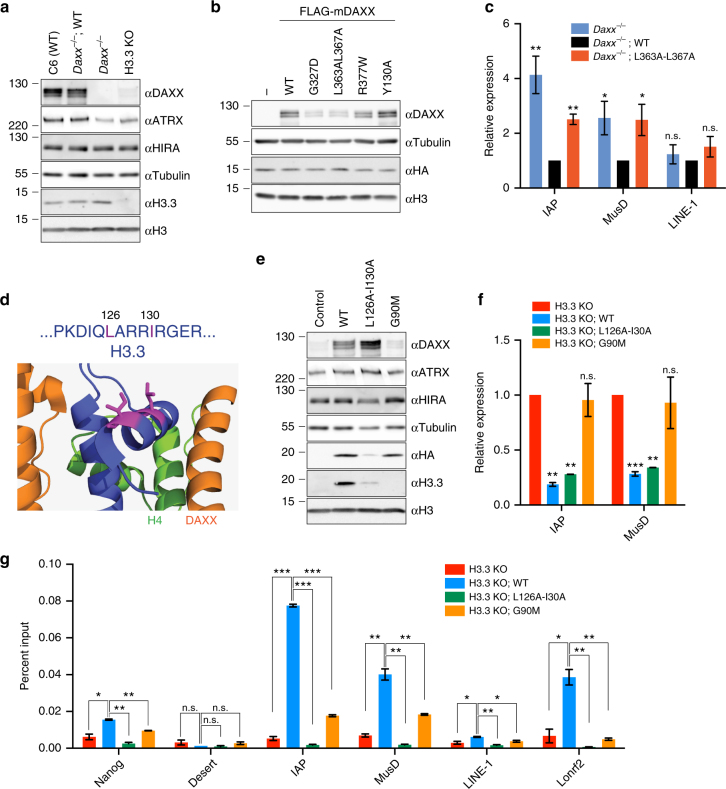
H3.3 stabilizes DAXX protein and affects ERV repression independently of its deposition. **a** Immunoblot for DAXX, ATRX, HIRA, and H3.3 in whole-cell lysates from the noted mESC cell lines, and loading controls anti-Tubulin and anti-H3. **b** Immunoblot of the indicated *Daxx* transgenes introduced into *Daxx* ^−/−^ mESCs, levels of HA-tagged endogenous H3.3B, and anti-Tubulin and anti-H3 loading controls. **c** Expression of IAP, MusD, and LINE-1 elements determined by RT-qPCR in *Daxx* ^−/−^ mESCs, rescued with *Daxx* transgenes (WT, L363A–L367A). Data are shown as relative expression and were normalized to *Gapdh* or *Actb*. Shown are results from four independent experiments (error bars depict S.E.M.). **d** Sequence context and location of amino acids L126 and I130 (highlighted in magenta) in H3.3 in a co-crystal structure in complex with DAXX and H4 (PDB ID: 4H9N). **e** Immunoblot of DAXX, ATRX, and HIRA in H3.3 KO mESCs (control) and cells stably transduced with the indicated H3.3-HA-FLAG transgenes. Immunoblots for DAXX, HIRA, ATRX, Tubulin, HA, H3.3, and H3 are shown. **a** and **e** were split from the same original image. The full blot is shown in Supplementary Fig. 10a. The H3.3 antibody was raised against the ‘Ala87-Ala88-Ile89-Gly-90’ motif in H3.3 and does therefore not recognize H3.3 G90M. **f** Expression of IAP and MusD elements determined by RT-qPCR in H3.3 KO mESCs and cells transduced with the indicated H3.3 transgenes. Data are shown as relative expression and were normalized to *Actb* from two independent experiments (error bars depict S.E.M.). **g** Anti-HA ChIP-qPCR at the indicated genomic regions for different H3.3-HA-FLAG transgenes in the background of H3.3 KO mESCs. Data are presented as per cent input (error bars depict S.E.M.). Shown are the results of two independent experiments. Asterisks in **c**, **f** and **g** denote statistical significance as obtained by Student’s *t*-test: *p < 0.05, **p < 0.01, ***p < 0.001, n.s. not significant

The unexpected result that ATRX is not required for DAXX-dependent repression of ERVs led us to investigate the role of histone H3.3 in this process. We assessed the relative levels of DAXX and histone H3.3 proteins in WT, *Daxx* ^−/−^, and H3.3 KO mESCs. As expected, H3.3 was absent in H3.3 KO mESCs and was not affected by presence or absence of DAXX (Fig. 4a). In addition, we found identical DAXX levels between WT and *Daxx* ^−/−^ mESCs expressing a WT transgene, indicating that our experiments were performed at physiologically relevant DAXX concentrations. Surprisingly, steady-state levels of DAXX were severely diminished in H3.3 KO mESCs (Fig. 4a). To confirm this result in a different genetic background, we treated WT mESCs with siRNAs directed against *H3f3a + b* or *Daxx*. Immunoblot analysis of these samples showed that knockdown of H3.3 resulted in decreased levels of DAXX protein, comparable to the knockdown of *Daxx* itself (Supplementary Fig. 7a, b).

To further investigate the link between DAXX and histone H3.3 protein levels, we studied DAXX mutants that are unable to interact with the H3.3–H4 heterodimer. One of those mutants, L357A–L361A (and its mouse analog L363A–L367A) was identified in a mutagenesis screen using recombinant DAXX expressed in insect cells via a baculovirus expression system (Supplementary Fig. 8a, b). The other two DAXX mutants, G327D and R377W, are murine analogs of mutations found in human malignancies^25^. All three DAXX mutants failed to interact with histones H3.3 and H4, however, interaction between the DAXX mutants with KAP1 was unaffected (Supplementary Fig. 8c). Interestingly, all histone binding-deficient DAXX mutants exhibited decreased steady-state levels as compared to WT DAXX or ATRX binding-deficient DAXX, both when ectopically expressed in *Daxx* ^−/−^ as well as in WT mESCs (Fig. 4b; Supplementary Fig. 8d, e), despite having equal mRNA levels in cells (Supplementary Fig. 8f). Additionally, the histone-binding DAXX mutant L363A–L367A exhibited de-repression of and loss of H3.3 at ERVs (Fig. 4c; Supplementary Fig. 8g). Taken together, these results indicate that the level of DAXX protein in cells is largely affected by its ability to interact with histones H3.3–H4.

The surprising finding that the absence of H3.3 decreases DAXX protein led us to hypothesize that low DAXX levels, rather than H3.3 deposition at LTRs, is the ultimate cause of ERV de-repression in the H3.3 KO mESCs^18^. To test this proposition, we designed a mutant H3.3 that could interact with DAXX, but could not be stably deposited into nucleosomes. A previous study investigated the effects of amino acid substitutions in H3 on the thermodynamic stability of the H3–H3ʹ interface within the symmetric (H3–H4)_2_ tetramer—an essential intermediate toward nucleosome formation^36^. The authors found that substitutions at positions L126 and L130 in yeast H3 led to disruption of hydrophobic interactions with the adjacent H3 molecule within the same nucleosome. Our previously described co-crystal structure of human DAXX with H3.3–H4 heterodimer show that L126 and I130 of histone H3.3 protrude into solvent and are not involved in DAXX binding (Fig. 4d). In a series of experiments, we confirmed that the L126A-I130A mutant H3.3 was not incorporated into nucleosomes, but still maintained interaction with DAXX (Supplementary Fig. 9). For the first set of experiments, WT, L126A-I130A, and a DAXX binding-deficient G90A histone H3.3 were expressed in MEFs. We found similar levels of WT and G90A histone H3.3, compared to very low levels of the L126A-I130A mutant in whole-cell immunoblots (Supplementary Fig. 9a). This result hinted that the L126A-I130A mutant H3.3 was not incorporated into chromatin—the predominant repository of cellular histones. To confirm this finding, we performed immunoprecipitation of the transgenic H3.3 from soluble nuclear extract of these cells, while carrying out acid extraction of deposited histones on the remaining chromatin pellet (Supplementary Fig. 9b). The L126A-I130A mutant co-immunoprecipitated with DAXX and histone H4, and was present at higher levels in the soluble fraction of the nuclear extract as compared to WT and G90A (Supplementary Fig. 9c). Conversely, immunoblots of acid extracted histones from the chromatin pellet confirmed that the H3.3 L126A-I130A mutant was not incorporated into chromatin (Supplementary Fig. 9d). In a second set of experiments, we immunoprecipitated mononucleosomes from MEFs expressing transgenic H3.3 WT, L126A-I130A, and the DAXX-binding mutant G90M (Supplementary Fig. 9e–g). Immunoblots of immunoprecipitated transgenic histones indicated that WT and G90M histone H3.3 were present in mononucleosomes (containing all four histone core species). However, the L126A-I130A mutant immunoprecipitated only DAXX and H4, and not H2A, H2B, or endogenous H3.3 (Supplementary Fig. 9h–j). We conclude from these experiments that the L126A-I130A histone H3.3 assembles with DAXX and histone H4 to form pre-deposition complexes, but is not stably incorporated into chromatin.

Immunoblot analysis of H3.3 KO mESCs transduced with WT or L126A-I130A histone H3.3 (as epitope-tagged transgenes) confirmed that DAXX steady state levels were stabilized by both the WT and deposition mutant H3.3 histones (Fig. 4e; Supplementary Fig. 10a, b). Moreover, both WT and L126A-I130A H3.3 were able to repress ERV expression in the background of H3.3 KO mESCs (Fig. 4f). Conversely, the DAXX binding-deficient H3.3-mutant G90M did not stabilize DAXX protein levels nor did it repress ERVs (Fig. 4e, f). Furthermore, ChIP-qPCR experiments for H3.3-HA confirmed that H3.3 L126A-I130A was not deposited at the LTRs of ERVs (Fig. 4g). Based on these data, we conclude that ERV repression is dependent on physiological normal levels of DAXX, but not upon deposition of H3.3 into nucleosomes.

**Fig. 5:**
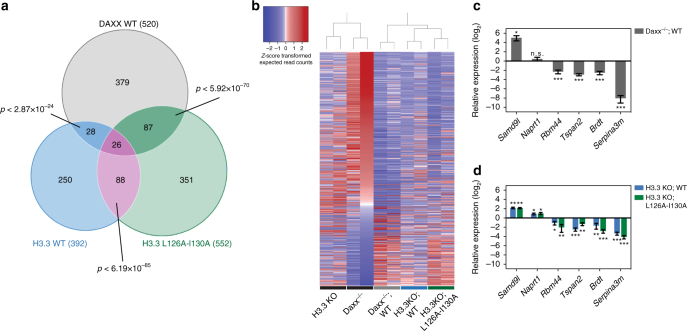
Analyses of DAXX-dependent and histone H3.3-dependent gene expression in mESCs. **a** Venn diagram depicting the intersection of differentially expressed genes (greater than twofold change, FDR > 5%) in DAXX WT and H3.3 WT/L16A-I130A compared to the respective KO cell line. Duplicate biological replicates for each cell line were analyzed in the RNA-Seq experiment. The *p*-values were determined by using hypergeometric distribution. **b** Heatmap showing gene expression pattern and hierarchical clustering of the indicated mESC lines with respect to genes differentially expressed (greater than twofold change) between mESC *Daxx* ^−/−^ and mESC *Daxx* ^−/−^;WT. Biological replicates (columns) are ordered according to hierarchical clustering of differentially expressed genes. The vertical listing of genes was based on descending *Z* scores for a *Daxx* ^−/−^ RNAseq replicate sample. **c**, **d** qRT-PCR validation of the RNA-Seq experiment for the indicated genes in *Daxx* ^−/−^ mESCs rescued with WT DAXX (**c**) or in H3.3 KO mESCs rescued with the indicated H3.3 transgenes (**d**). Data are shown as relative expression and were normalized to *Actb*. Shown are results from four independent experiments (error bars depict S.E.M.). Asterisks denote statistical significance as obtained by Student’s *t*-test: *p < 0.05, **p < 0.01, ***p < 0.001, n.s. not significant

The finding that histone H3.3 influences DAXX-dependent repression by ERVs through stabilizing steady-state DAXX protein levels led us to explore if H3.3 also affects DAXX-dependent gene regulation. To this end, we used RNA-Seq to identify DE genes (>2.0 fold-change) in *Daxx* ^−/−^ and H3.3 KO mESCs rescued with DAXX and H3.3 transgenes. We found that 27% of DAXX DE genes (141 out of 520) were also DE in the presence of either WT or L126A-I130A histone H3.3 (Fig. 5a–c; Supplementary Table 2). The majority of these genes (113 genes or 80%) were dependent on expression of the L126A–L130A mutant or WT histone H3.3, while only 28 genes (20%) were dependent only on WT histone H3.3. These data suggest that histone H3.3 levels influence more than a quarter of DAXX-dependent DE genes in mESCs. Additionally, the finding that the gene expression profile of the non-depositable mutant histone overlapped with DAXX better than the WT histone suggests that stabilization of DAXX protein levels, rather than deposition into nucleosomes, is the dominant mechanism by which histone H3.3 influences DAXX-dependent gene expression in mESCs.

**Fig. 6:**
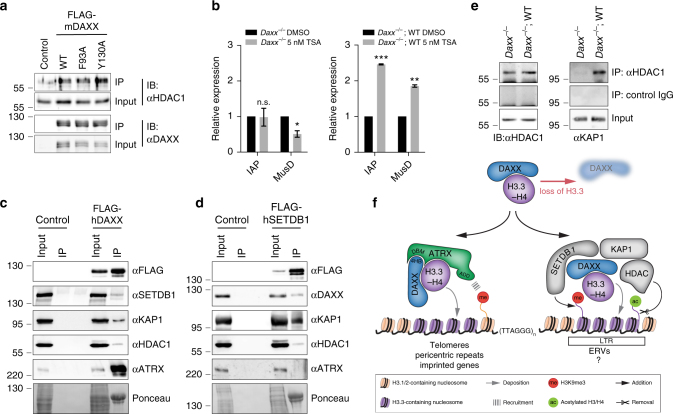
HDAC activity contributes to DAXX-mediated ERV repression. **a** Immunoblot for DAXX and HDAC1 in input and eluate from immunoprecipitation of FLAG-mDAXX (WT, F93A, and Y130A) and control (no FLAG-tagged transgene) in *Daxx* ^−/−^ MEFs. **b** Expression of IAP and MusD elements determined by RT-qPCR *Daxx* ^−/−^ and *Daxx* ^−/−^;WT treated with DMSO control or 5 nM trichostatin A (TSA) for 48 h. Data are presented as relative expression and were normalized to *Actb*. Shown are results from two independent experiments (error bars depict S.E.M.). Asterisks denote statistical significance as obtained by Student’s *t*-test: *p < 0.05, **p < 0.01, ***p < 0.001, n.s. not significant. **c** 293T cells transfected with no construct (control) or FLAG-hDAXX were subjected to anti-FLAG IP. **d** 293T cells transfected with no construct (control) or FLAG-hSETDB1 were subjected to anti-FLAG IP. Inputs and elutions in **c** and **d** were analyzed for SETDB1, DAXX, ATRX, HDAC1, and KAP1 by immunoblot. Ponceau stains show the overexpressed FLAG-tagged proteins present in the elution samples. **e** Immunoprecipitation for endogenous HDAC1 alongside IgG control was performed in *Daxx* ^−/−^ and *Daxx* ^−/−^;WT MEF. Shown are immunoblots for HDAC1 and KAP1 in input and eluates. **f** At least two biochemically distinct DAXX–H3.3-containing complexes exist in mammalian cell: One complex contains DAXX, ATRX, and H3.3–H4, and deposits H3.3-containing nucleosomes at telomeres, and is involved in regulating pericentric repeats and imprinted genes. The second complex contains DAXX, H3.3–H4, SETDB1, and KAP1 proteins and functions to repress ERVs. The DAXX–SETDB1–KAP1 complex may have evolved other functions in addition to repression of ERVs

Previous work implicated HDACs in the maintenance of repressed chromatin states at ERVs^19, 37^. Furthermore, DAXX has been noted to associate with HDACs for the repression of gene expression^14, 38, 39^. We found that HDAC1 co-immunoprecipitated with both WT and ATRX binding-deficient DAXX in MEF nuclear extract (Fig. 6a). Therefore, we assessed ERV expression upon treatment with the lysine deacetylase inhibitor trichostatin A (TSA) in the presence and absence of DAXX. Incubation with TSA led to upregulation of IAP (2.5-fold) and MusD (1.8-fold) in *Daxx* ^−/−^ mESCs rescued with a WT *Daxx* transgene (Fig. 6b). However, TSA treatment in the absence of DAXX did not cause additional de-repression of IAP or MusD elements. These results suggest that DAXX may help repress ERVs through recruitment of lysine deacetylase activity, rather than through H3.3 deposition activity. We found that HDAC1 also associates with SETDB1 in co-immunoprecipitation experiments (Fig. 6c, d). Additionally, endogenous HDAC1 co-immunoprecipitated KAP1 from MEF nuclear extracts in a DAXX-dependent manner (Fig. 6e). DAXX may therefore serve as a bridging factor between the transcriptional repressing activities of HDACs and the H3K9me3-directed activity of the SETDB1–KAP1 proteins.

## Discussion

In this study we present a high-resolution structure of an interaction surface between domains found in ATRX and DAXX. Furthermore, we demonstrate that these domains are necessary to mediate ATRX–DAXX interactions in vitro and in vivo. Our gene expression studies revealed that mutations in the DAXX 4HB that disrupt the ATRX–DAXX interaction phenocopy the genetic loss of *Daxx* with respect to ATRX–DAXX-regulated genes. The high frequency of inactivating mutations of *DAXX* and *ATRX* genes in human tumors points to tumor suppression activities of this complex. We showed that a tumor-derived DAXX mutation has substantially weakened binding affinity for ATRX, a finding that underscores the likely need for ATRX–DAXX complex formation in suppression of tumorigenesis.

Our structural and biochemical studies led to the identification of two functionally and mechanistically distinct DAXX–H3.3–H4-containing complexes involved in the maintenance of repressed chromatin states (Fig. 6f). One complex composed of ATRX, DAXX, and histones H3.3–H4 functions in the assembly of H3.3-containing nucleosomes at pericentric and telomeric repeats (Fig. 6f, left). The protein partners are present at equal stoichiometries within the ATRX–DAXX–H3.3–H4 complex^9, 11^. A second complex containing DAXX, histones H3.3–H4, SETDB1, KAP1, and HDAC activity in unknown stoichiometries helps facilitate repression of ERVs in mESCs (Fig. 6f, right).

Our biochemical experiments revealed that SETDB1 does not associate with ATRX, and that DAXX 4HB mutations that disrupt interaction with ATRX have no effect on DAXX association with SETDB1 and KAP1. These data suggest that ATRX and SETDB1–KAP1 interact with different surfaces of DAXX. However, the mutually exclusive nature of ATRX and SETDB1–KAP1 interactions hints at the possibility of overlapping interaction surfaces on DAXX. Our finding that association between DAXX and SETDB1–KAP1 is not dependent on histones H3.3–H4 suggests that the key interaction domain is neither the 4HB nor the HBD. A previous study proposed that the first fifty residues and the entire C-terminal half of DAXX are intrinsically disordered^33^ and could serve to mediate protein-protein interactions. Alternatively, the N-termini and C-termini of DAXX contain highly conserved SUMO-interacting motifs (SIM) (Fig. 1a), and a recent study showed that the C-terminal SIM of DAXX was important for its repressive function on IAP element transcription^17^. This result suggests that interaction between DAXX and sumoylated proteins is likely important for DAXX-mediated and SETDB1–KAP1-mediated repression of ERVs. In support of this model, auto-sumoylation of KAP1 by its intrinsic E3 SUMO ligase activity was found to be important for interaction with SETDB1^40, 41^.

Our previous structural study identified an unprecedented 4,500 Å^2^ of interaction surface between the H3.3–H4 heterodimer and the DAXX HBD^11^. Two pieces of data in our study point to H3.3 as a critical factor in modulating DAXX protein levels: the depletion of H3.3, or amino acid substitutions in DAXX that abrogate binding to H3.3–H4 lead to loss of DAXX protein levels. The DAXX HBD does not establish a stable conformation in the absence of H3.3–H4 binding^42^, and we speculate that H3.3–H4 may help the DAXX HBD adopt a conformation that promotes DAXX protein stabilization in cells. Additionally, we demonstrate that steady-state levels of ATRX are modulated through interaction with DAXX in both mESCs and somatic cells. This finding suggests that levels of DAXX protein may influence many ATRX-dependent cellular processes and phenotypes.

Chromatin-based mechanisms such as 5-methylcytidine and histone post-translational modifications aid in the repression of ERVs. Recruitment by KAP1 and catalysis of the H3K9me3 by SETDB1 has been shown to be essential for proviral repression in embryonic stem cells^19, 20^. A recent study suggested that histone H3.3 deposited at ERVs by ATRX–DAXX is required for SETDB1-mediated H3K9me3 repression^18^. In our study, we confirmed that DAXX is necessary for repression of ERVs, however, we do not observe de-repression upon the loss of ATRX or by the disruption of the ATRX–DAXX complex through single point mutations. Accordingly, we found that DAXX-dependent deposition of H3.3 at the LTRs of ERVs also occurs independent of ATRX. Furthermore, we did not find any evidence for association between ATRX and SETDB1–KAP1 co-repressor proteins. Our experiments using an H3.3 mutant that cannot be deposited into chromatin, yet still retains ERV repressive activity suggests that H3.3-containing nucleosomes are not required for ERV repression. Instead, we found that H3.3 plays an indirect role in the repression of ERVs through its stabilization of DAXX.

We show that the deposition of histone H3.3 at ERVs is dispensable for DAXX–SETDB1–KAP1-mediated repression, and that physiological levels of H3.3 are required to maintain DAXX protein levels. This unexpected role for histone H3.3 demonstrates a function for a histone variant in a non-nucleosomal context. Additionally, the surprising discovery of the interplay between H3.3 and levels of DAXX and ATRX proteins raises important questions regarding phenotypes attributed to nucleosomal H3.3 from gene KO studies. Previous studies have implicated histone H3.3 deposition by HIRA or ATRX–DAXX in various developmental transitions including fertilization, pluripotency, as well as terminal differentiation of cells^43^. It is uncertain whether these phenotypes are attributable to H3.3 incorporation into nucleosomes or the described deposition-independent function of H3.3 in the stabilization of DAXX and ATRX protein levels.

We demonstrate that DAXX-dependent HDAC activity plays an important role in maintaining ERV repression. The details of the interplay of DAXX, HDACs, and SETDB1–KAP1 in the process of ERV repression will need to be addressed in future studies. DAXX is reported to interact with many nuclear proteins^44^. Perhaps other genomic loci or developmental contexts, DAXX may serve as an adaptor or bridging factor for additional non-HDAC proteins to promote silent chromatin states. While SETDB1–KAP1 co-repressor proteins are reportedly unnecessary for ERV repression in differentiated somatic cells^20^, it is noteworthy that interaction between DAXX and SETDB1–KAP1 is detectable in mESCs and in somatic cell lineages such as MEFs, 293T, and HeLa S3 cells. The ERVs repressed by DAXX and SETDB1–KAP1 in mESCs are only found in rodents, however, a very recent report showed that KAP1 regulates gene expression in human neural progenitor cells by establishing heterochromatin formation at primate-specific ERVs^45^. It will be important to explore whether the DAXX–SETDB1–KAP1–HDAC1 complex plays a role in repressing ERVs in humans.

## Methods

### Protein expression and purification

The human DBM (residues 1265–1288) of ATRX was directly fused to the C terminus of the 4HB (residues 50–144) of human DAXX without any linker residue. The resulting DAXX 4HB-ATRX DBM expression cassette was cloned into a modified RSFDuet-1 vector (Novagen), with an N-terminal His_6_-SUMO tag, and was expressed in BL21(DE3)-RIL cell strain (Stratagene). The expressed fusion protein was first purified on a Ni-NTA affinity column. After removing the His_6_-SUMO tag by using Ulp1 (SUMO protease), the protein was further purified with a heparin column and HiLoad 16/600 Superdex 200 column (GE Healthcare). The DAXX 4HB alone was also cloned into a modified RSFDuet-1 vector (Novagen). All the mutations on DAXX 4HB were introduced by standard site-directed mutagenesis. The WT DAXX 4HB or its mutants were expressed and purified in a similar way as above.

### Crystallization

The DAXX 4HB—ATRX DBM fusion protein at 26 mg ml^−1^ was crystallized under conditions of 0.1 M sodium cacodylate, pH 6.8, 1.2 M ammonium sulfate and 3% 1,5-diaminopentane dihydrochloride, using sitting-drop vapor-diffusion method at 20 °C. In this process, 0.5 µl of protein were mixed with 0.5 µl of mother liquor. All crystals were soaked in a cryoprotectant made from mother liquor supplemented with 25% glycerol before flash freezing in liquid nitrogen.

### Structure determination

The data set for the DAXX 4HB—ATRX DBM fusion protein was collected at 0.979 Å on 24-ID-C NE-CAT (Advanced Photo Source, Argonne National Laboratory). The data was processed by using the HKL 2000 program. The initial structure for the DAXX 4HB–ATRX DBM fusion protein was solved by molecular replacement in PHASER with an NMR derived structure of DAXX 4HB (Protein Data Bank (PDB) ID: 2KZS) as a search model and manually refined and built using Coot. The final structure was refined to 3.10 Å resolution using PHENIX. The Ramachandran plot showed 90.2% favored, 9.7% allowed, and 0.1% outliers. Supplementary Table 1 summarizes the statistics for data collection and structural refinement.

### Isothermal titration calorimetry

All the ITC titrations were performed on a Microcal ITC 200 calorimeter at 20 °C. The ATRX DBM WT or mutant peptides were all synthesized at Tufts University Peptide Core Facility. The exothermic heat of the reaction was measured by 17 sequential 2.2 μl injections of the peptides (1.5 mM in buffer 20 mM Tris pH 7.5 and 0.3 M NaCl) into 200 µl solution of the DAXX 4HB or its mutants (150 µM in the same buffer), spaced at intervals of 180 s. The data were processed with Microcal Origin software and the curves were fit to a single site-binding model.

### GST pulldown from MEF nuclear extract

GST-tagged murine DAXX 4HB (residues 60–149) and ATRX DBM (residues 1230–277) were cloned into pGOOD vector (modified version of pGEX6p1) and expressed in BL21(DE3) cells via induction with 1 mM IPTG. GST only, GST-DAXX 4HB (WT and Y130A), and GST-ATRX DBM (WT and L1255A) were purified using Glutathione Sepharose 4B (GE Healthcare Life Sciences) using 20 mM glutathione in the elution, followed by dialysis into 20 mM HEPES-KOH pH 7.9, 200 mM KCl, 1 mM EDTA, 0.8 mM PMSF, 5 mM 2-mercaptoethanol, 2 mM DTT, and 10% glycerol. MEF nuclear extract (at 200 mM KCl) was incubated with GST-tagged proteins coupled to glutathione beads at 4 °C for 4 h. Beads were washed three times for 5 min with wash buffer (same as dialysis buffer but with 350 mM KCl and 0.01% NP-40) and then transferred into micro spin columns. GST-tagged proteins and bound proteins were eluted with elution buffer (same as wash buffer, supplemented with 20 mM glutathione, pH adjusted to 8.0).

### Immunoblot

Immunoprecipitation samples or whole-cell lysates were separated by SDS-PAGE, transferred to nitrocellulose membrane, blocked in 5% non-fat milk in Tris-buffered saline with 0.5% Tween-20, probed with primary antibodies, and detected with horseradish peroxidase-conjugated anti-rabbit or anti-mouse secondary antibodies (Bio-Rad). A list of the antibodies used is provided in Supplementary Table 3. Uncropped images for all immunoblot and gel images are provided in Supplementary Fig. 11.

### ES cell lines

The C6 line was derived from B6(Cg)-Tyrc-2J/J C57BL/6J background mice, the *Daxx* ^−/−^ cell line was provided by Philip Leder^46^, *Atrx* ^fl^, and *Atrx* ^del^ mESCs were provided by David Picketts^47^. The H3.3 K.O. cells was derived from a mixed 129 × C57BL/6J background and provided by David Allis^48^. The *Setdb1* ^fl/fl^ cell line was provided by Yoshi Shinkai^20^. The *Kap1* ^fl/fl^ cells were provided by Didier Trono^19^.

### ES cell culture

mESCs were maintained under 2i + LIF conditions in a medium containing a 1:1 mix of Dulbecco’s modified Eagle’s medium (DMEM)/F12 (Life Technologies 11330-033) and Neurobasal medium (Life Technologies 21103-049) with the addition of N2 and B27 supplements (Life Technologies 17502-048 and 17502-044, 1:100 dilutions), penicillin/streptomycin (100 U ml^−1^ and 100 µg ml^−1^, respectively; Life Technologies 15140-122), 0.1 mM 2-mercaptoethanol, 1× GlutaMAX^TM^-I (Life Technologies 35050-061), 1× MEM NEAA (non-essential amino acids; Life Technologies 11140-050), Leukemia inhibitory factor (LIF), CHIR99021 at 3 μM (TOCRIS) and PD0325901 at 1 μM (Selleckchem). All experiments were performed under feeder-free conditions on dishes coated with 0.1% bovine gelatin. Cells were tested regularly for mycoplasma using the e-Myco^TM^ Mycoplasma PCR Detection Kit (iNtRON Biotechnology). For CRE-ERt mediated gene KO in *Setdb1* ^fl/fl^ and *Kap1* ^fl/fl^ mESCs, cells were treated with 800 nM 4-hydroxytamoxifen for 4 days.

### Immortalized cell lines

HeLa S3 (ATCC CCL-2.2), 293T (ATCC CRL-3216), and MEFs (*Daxx* ^−/−^ and WT) were provided by Philip Leder (Michaelson 1999 G&D), and were maintained in DMEM supplemented with 1× GlutaMAX^TM^, penicillin/streptomycin, and 10% fetal bovine serum (Gibco).

### Lentiviral plasmid construction and lentivirus preparation

FLAG-HA-tagged H3.3 and FLAG-tagged murine DAXX (mDAXX) were cloned into pCDH-EF1-MCS-Puro lentiviral vectors (System Biosciences). Standard site-directed mutagenesis techniques were used to generate missense mutations in both H3.3 and mDAXX. To produce lentivirus, 293T cells underwent transfection with lentiviral vector and helper plasmids (psPAX2 and pMD2.G). Supernatant containing lentivirus was collected, filtered, and concentrated after 72 h.

### Lentiviral transduction

To generate cell lines stably expressing various H3.3, mDAXX, or shRNA constructs, cells were transduced with concentrated lentivirus in the presence of 5 µg ml^−1^ Polybrene^®^ (Santa Cruz Biotechnology sc-134220). Transduced cells were grown under puromycin selection (4 µg ml^−1^ for H3.3 transgenes; 1.25 µg ml^−1^ for mDAXX transgenes; 2 µg ml^−1^ for shRNAs) 48 h after transduction. Cells were collected for experiments within 1–3 weeks after lentiviral transduction.

### Transient transfection

293T cells at 50–60% confluence were transfected with 20 µg of pCMV2-FLAG-hSETDB1 or pRK5-FLAG-hDAXX in Opti-MEM^®^ I Reduced Serum Media (Life Technologies 31985-070) using 60 µl Lipofectamine^®^ 2000 (Invitrogen 11668-00). Media was replaced with full growth media 12 h after transfection. Cells were collected for co-immunoprecipitation experiments 48 h after transfection.

### Treatment of mESCs with trichostatin A

For global inhibition of HDAC class I and II mediated deacetylation, 5 × 10^5^ mESCs were seeded per well of a 12-well plate in 1 ml 2i + LIF media. On the next day, cells were treated with 5 nM TSA alongside DMSO only control and collected for analysis after 48 h of treatment.

### Reverse transcription and quantitative PCR

Whole-cell RNA was extracted using a Quick-RNA™ MiniPrep kit (Zymo) or a RNeasy Mini kit (Qiagen), digested with DNase I off-column, followed by a second column clean-up. cDNA was prepared from 1 µg RNA using the SuperScript VILO cDNA Synthesis Kit (Thermo Fisher; random hexamer primers). Quantiative real-time PCR was performed on diluted cDNA in the presence of 0.5 µM of forward and reverse primers using Power SYBR^®^ Green Master Mix. Program: Initial 95 °C for 10′, then 95 °C for 20″, 60 °C for 30″, 72 °C for 20″ (52 cycles). A list of the quantitative PCR primers used is provided in Supplementary Table 4.

### Native chromatin immunoprecipitation

Per sample, 20 × 10^6^ murine embryonic stem cells were washed once with 1 ml of PBS (+ 2 mM MgCl_2_). Cells were resuspended in digestion buffer (50 mM Tris-HCl pH 7.6, 1 mM CaCl_2_, 0.4% Triton X-100, 1× protease inhibitor cocktail, 0.4 mM PMSF) and chromatin was subsequently fragmented by incubation with monococcal nuclease at 37 °C for 5 min. Reactions were stopped by adding EDTA and EGTA at final concentrations of 5 mM and 1.67 mM, respectively. Samples were further sonicated using a S220 Covaris focused ultrasonicator for 4 × 60 s (with 30 s breaks in between) at peak power 170, duty factor 10, and 200 cycles per burst. Samples were then dialyzed against RIPA buffer (10 mM Tris pH 7.6, 1 mM ETDA, 0.1% SDS, 0.1% sodium deoxycholate, 1% Triton X-100, 0.5 mM PMSF) for 2 h at 4 °C. Chromatin in supernatant was incubated with 4 µg of anti-HA antibody overnight. After incubation, 40 μl of Dynabeads M-280 (anti-mouse, Life Technologies) were added and incubated for 6 h at 4 °C. Beads were then washed three times in RIPA buffer, two times in RIPA + 0.3 M NaCl, two times in RIPA + 0.25 M LiCl, and once in TE (10 mM Tris pH 8.0, 1 mM EDTA) buffer + 50 mM NaCl. Bound chromatin was eluted in ChIP elution buffer (50 mM Tris-HCl pH 8.0, 10 mM ETDA, 1% SDS) by heating at 65 °C for 30 min. DNA was isolated by proteinase K digestion, followed by silica-membrane column purification (Gel/PCR DNA Fragment Extraction Kit, IBI Scientific).

### Purification of HeLa DAXX-associated complexes

HeLa S3 nuclei from 2 × 10^9^ cells were prepared by hypotonic lysis and extracted with 400 mM ammonium sulfate. Dialyzed nuclear extracts (250 mM KCl) were centrifuged for 10 min at 23,500 × *g* at 4 °C. Supernatants were incubated with 300 µl of packed anti-FLAG M2 beads (Sigma) for 4 h at 4 °C. After incubation, beads were transferred into a gravity flow column and washed with 10 volumes of wash buffer (20 mM HEPES-KOH pH 7.9, 200 mM KCl, 1 mM EDTA, 10% glycerol, 0.01% NP-40, 1 mM 2-mercaptoethanol, 0.2 mM PMSF) by gravity flow. FLAG-DAXX-containing complexes were eluted in 250 µl fractions of elution buffer (wash buffer supplemented with 0.3 mg ml 3×FLAG peptide). Elutions fractions containing FLAG-DAXX were pooled and adjusted to 150 mM KCl by addition of buffer A (20 mM HEPES-KOH pH 7.9, 1 mM EDTA, 0.01% NP-40, 10% glycerol). Pooled fractions were loaded onto a Mono Q PC 1.6/5 column (GE Healthcare; column volume: 0.101 ml) and separated by a gradient from 15% buffer B (20 mM HEPES-KOH pH 7.9, 1 M KCl, 1 mM EDTA, 0.01% NP-40, 10% glycerol) to 100% buffer B (gradient length: 20 column volumes). Fractions of 150 µl were collected.

### Anti-FLAG co-immunoprecipitation

Lysate from 2.5 × 10^7^ 293T cells (with transient transgenes) or from 4 × 10^7^ MEFs (with stable transgenes) was prepared by resuspension in a total of 1.5 ml lysis buffer (20 mM HEPES-KOH pH 7.9, 300 mM KCl, 1 mM EDTA, 0.12% Triton X-100, 2 mM 2-mercaptoethanol, 0.4 mM PMSF, 2× protease inhibitor cocktail), followed by douncing and separation of the insoluble fraction by centrifugation. Per sample, 40–60 µl of packed anti-FLAG M2 beads (Sigma) were added to the lysate and incubated rotating at 4 °C for 3 h. Incubation was performed in the presence of 50 µg ml^−1^ ethidium bromide. Beads were washed three times with Wash buffer (same as lysis buffer but omitting 2-mercaptoethanol) for 5 min. Finally, beads were transferred into microspin columns and samples were eluted with 30–60 µl elution buffer (wash buffer supplemented with 500 ng µl^−1^ 3×FLAG peptide; Tufts University Peptide Core Facility) via incubation for 5 min on ice and centrifugation at 300 × *g*.

### Immunoprecipitation for endogenous HDAC1

Lysate from *Daxx* ^−/−^ and *Daxx* ^−/−^;WT MEFs was prepared by resuspension in a total of 1.5 ml lysis buffer (20 mM HEPES-KOH pH 7.9, 300 mM KCl, 1 mM EDTA, 0.12% Triton X-100, 2 mM 2-mercaptoethanol, 0.4 mM PMSF, 2× protease inhibitor cocktail), followed by douncing and separation of the insoluble fraction by centrifugation. Per sample 50 µl of Dynabeads M-280 (anti-rabbit, Life Technologies) were washed briefly with PBS + 0.01% NP-40, then incubated with 5 µg of primary antibody (anti-HDAC1 or IgG control) for 1 h in PBS supplemented with 50 µg ml^−1^ BSA. Beads were washed and then incubated with the prepare lysates for 3 h at 4 °C. Beads were washed three times with wash buffer (20 mM HEPES pH 7.9, 250 mM KCl, 1 mM EDTA, 0.12% Triton-X100, 0.4 mM PMSF) for 2 min at 4 °C. Immunoprecipitated proteins were eluted by incubation with Elution buffer (70 mM glycine pH 2.5, 150 mM NaCl) for 5 min at RT.

### Analysis of RNA-Seq data

PolyA + RNA-Seq data sets for ATRX and DAXX KO mESCs available on Gene Expression Omnibus (GEO; accession number: GSE73881) were used in this study. RNA-Seq data was aligned to the reference murine genome using Bowtie and expression of transcripts was analyzed using RSEM with default parameters^49^. DE genes were identified using EBseq and genes showing a posterior probability of differential expression >0.95 and a fold change greater or equal to 5 folds in either direction were shortlisted for the downstream analysis^50^.

For the data sets described in Fig. 5, read mapping, transcript assembly, and expression estimation were performed as described for ATRX and DAXX KO mESCs data sets. DE genes were identified using EBSeq. Genes were classified as DE if (1) they exhibited a posterior probability of differential expression > 0.95 with a FDR < 0.05 and (2) a fold change greater or equal to 2 in either direction in comparison to the respective KO cell line. Gene expression pattern across all the samples was analyzed for the DAXX DE genes (520). A *Z*-score transformation was performed across rows on the data matrix consisting of normalized TPM counts obtained using a median normalization approach^51^. The genes were then sorted in descending order with respect to the TPM values in DAXX KO2 data set to generate the heatmap depicting the expression pattern across all the samples. A consensus dendrogram was generated from the Euclidean distances between median-normalized TPM values to assess the hierarchical clustering among all the samples.

### Sequence alignment

The sequence alignment comparing the ATRX DBM amino acid sequence across different vertebrate species was performed with MegAlign Pro (DNASTAR Lasergene), using the Clustal Omega algorithm.

### GST pulldown with ATRX DBM and untagged 4HB

Truncations of GST-tagged ATRX DBM were expressed in *Escherichia coli* and purified from *E. coli* lysate using Glutathione Sepharose 4B beads. Similar amounts of bead bound proteins were used for pulldown with purified DAXX 4HB protein. The pulldowns were performed with wash buffer containing 0.3 or 1.0 M NaCl, and the final samples were analyzed with SDS-PAGE stained with Coomassie Brilliant Blue.

### Radioactive in vitro histone methyltransferase assay

A total of 1.0 µg of C-terminally K-biotinylated H3^aa1–20^ peptide (Tufts University Peptide Core Facility) were incubated with 20 µl of FLAG-hDAXX Mono Q fractions for 1 h at 30 °C in a reaction buffer containing 50 mM HEPES-KOH pH 7.9, 0.5 mM DTT, 2 mM MgCl_2_, and 0.25 mM PMSF. A total of 2 µl of radioactive [^3^H]-labeled *S*-adenosyl methionine was present during the reaction. For quantification, reactions were spotted onto P81 nitrocellulose filters (Whatman), filters were washed for three times using 100 mM sodium bicarbonate, dipped into acetone, and air-dried. Samples were subsequently subjected to liquid scintillation counting for activity measurement.

### Lentiviral transduction with shRNAs and *Daxx* transgenes

To generate C6 mESCs stably expressing shRNA or mDAXX constructs, cells were transduced with concentrated lentivirus in the presence of 5 µg ml^−1^ Polybrene (Santa Cruz Biotechnology sc-134220). Cells were grown under puromycin selection (1.25 µg ml^−1^ for shRNAs, 0.5 µg ml^−1^ for *Daxx* transgenes) 48 h after transduction. Cells were collected for experiments within 2 weeks after lentiviral transduction. Sequences for shRNAs used are listed in Supplementary Table 5.

### siRNA knockdown

siRNAs were resuspended in 1× siRNA dilution buffer (60 mM KCl, 6 mM HEPES-KOH pH 7.5, 0.2 mM MgCl_2_) at 100 µM and 20 µM stock concentrations. Concentration was verified using absorbance readings at 260 nm. For siRNA transfection of mESCs, 3 × 10^5^ cells were seeded per well of a 12-well plate in 1 ml 2i + LIF media. Per well, cells were transfected with 2 µl lipofectamine and 50 pmol siRNA in 200 µl OPTI-MEM. After 12 h of transfection, media was replaced with fresh 2i + LIF media. Cells were collected for protein and RNA analysis 72 h after transfection. Sequences for siRNAs used are listed in Supplementary Table 6.

### FLAG-DAXX co-immunoprecipitation in mESCs

For FLAG-DAXX co-immunoprecipitation, 20–40 × 10^6^ mESCs were subjected to the same procedure as described in the main methods section.

### Acid histone extraction

Chromatin pellets after 0.4 M ammonium sulfate extraction were dissolved in 0.4 N H_2_SO_4_ by overnight rotation at room temperature. After centrifugation, supernatants were collected, proteins were precipitated by addition of 1/3 volume 100% TCA with 0.1% sodium deoxycholate on ice, washed twice with ice cold acetone, and resuspended in deionized water.

### Endogenous mononucleosome purification

MEF nuclei from 1 × 10^7^ were isolated using 15 ml of hypotonic buffer A (15 mM HEPES pH 7.9, 10 mM KCl, 5 mM MgCl_2_, 0.5 mM EDTA, 1 mM DTT, 0.8 mM PMSF, 1 mM benzamidine, 1 mM sodium metabisulfite) followed by centrifugation at 4000 rpm. Nuclei were resuspended in 6 ml of buffer AC (15 mM HEPES pH 7.9, 30 mM KCl, 3 mM CaCl_2_, 1 mM DTT, 0.4 mM PMSF, and 2× protease inhibitor cocktail) and incubated with Micrococcal Nuclease (MNase) (Worthington) at a final concentration of 25 U ml^−1^ for 20 min. The reaction was stopped by addition of 3 mM EGTA, 5 mM EDTA, 0.1% Triton-X100, and 150 mM KCl (final concentrations). The suspension was centrifuged at 14,000 rpm for 10 min and HA-FLAG-tagged H3.3-containing mononucleosomes were subsequently purified from the supernatant by incubation with 150 µl of M2 anti-FLAG resin (Sigma Aldrich) for 3 h at 4 °C. After washing twice with 150 mM NaCl and once with 300 mM NaCl in wash buffer (20 mM HEPES, 1 mM EDTA, 10% glycerol, 0.01% NP-40, 1 mM BME, 0.4 mM PMSF, 1 mM benzamidine, 1 mM sodium metabisulfite), mononucleosomes were recovered from the beads in elution buffer (wash buffer supplemented with 150 mM NaCl and 500 ng µl^−1^ 3×FLAG peptide).

### Statistical analysis

Statistical analysis was performed using Student’s *t*-test (two-sample *t*-test) unless stated otherwise.

### Data availability

The authors declare that the data supporting the findings of this study are available within the article and its Supplementary Information, or from the authors upon reasonable request. RNA-Seq data that support the findings of this study have been deposited in the GEO database with the primary accession code GSE102688 (https://www.ncbi.nlm.nih.gov/geo/query/acc.cgi?acc=GSE102688). Atomic coordinates and structure factors of the DAXX 4HB—ATRX DBM complex have been deposited in the PDB under primary accession code 5Y6O (http://www.rcsb.org/pdb/explore.do?structureId=5y6o).

## Electronic supplementary material

Supplementary Information
