# Supplementary material for: Structural and mechanistic insights into ATRX-dependent and -independent functions of the histone chaperone DAXX
Source: Nat Commun. 2017 Oct 30;8:1193. doi: 10.1038/s41467-017-01206-y (PMC5662737; doi:10.1038/s41467-017-01206-y)
Supplement: Supplementary file 1 — Supplementary Information [file 41467_2017_1206_MOESM1_ESM.pdf]

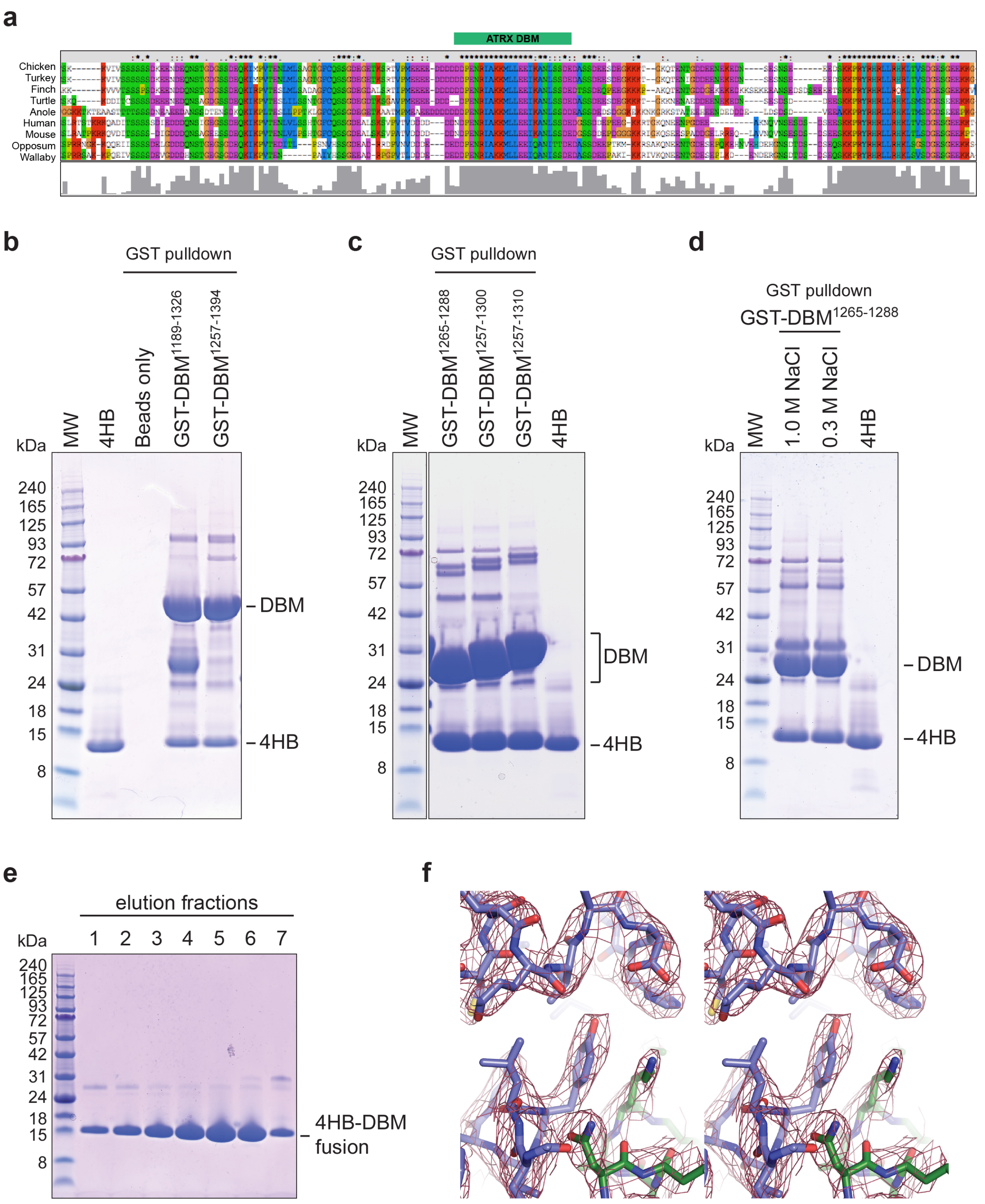

Supplementary Figure 1 | Identification of the minimal interaction surface between the DAXX 4HB and the ATRX DBM. (**a**) Alignment (ClustalX software) showing amino acid residues of the hATRX DBM and surrounding residues of ATRX homologs found in the indicated vertebrate species. (**b**) Coomassie gel of pulldown for hDAXX 4HB using hATRX GST-DBM of different length as bait. (**c**) Coomassie gel of GST pulldown for hDAXX 4HB using hATRX GST-DBM of different length as bait. (**d**) Coomassie gel of GST pulldown for hDAXX 4HB with GST-DBM$^{1265\text{-}1288}$ in the presence of 0.3 M and 1.0 M NaCl. (**e**) Coomassie gel showing the gel filtration profile of purified 4HB-DBM fusion protein. The most pure fractions were concentrated and used for crystallization. (**f**) Stereoview depicting a portion of the final electron density map (2Fo – Fc) with a contour level of 1σ.

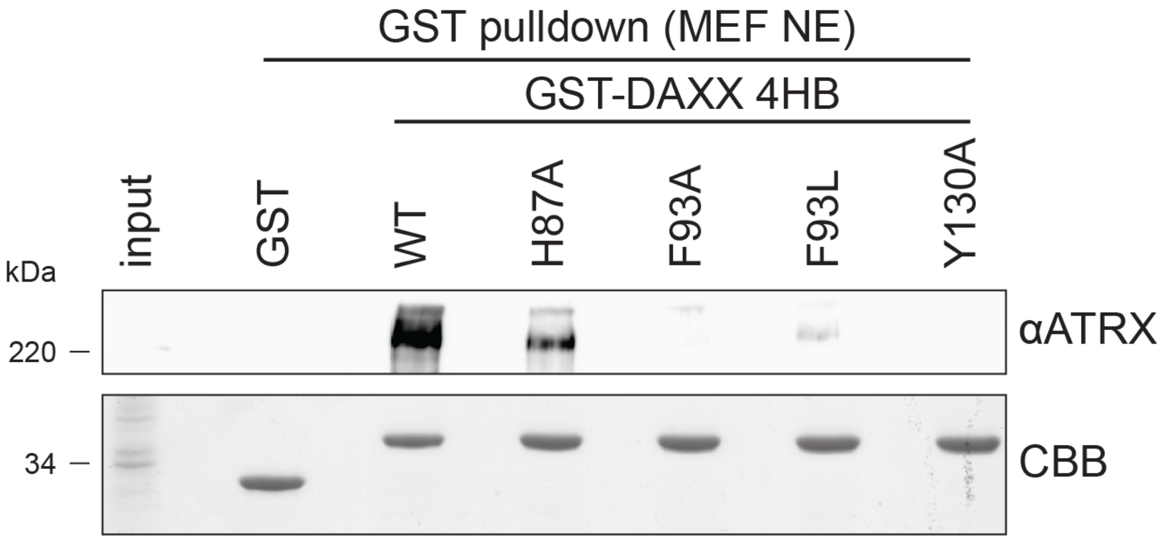

Supplementary Figure 2 | DAXX 4HB interacts with full-length ATRX. Pulldown of full-length ATRX from MEF nuclear extract using GST-tagged mDAXX 4HB (WT and indicated mutants) as bait. Immunoblot with anti-ATRX antibody is shown. Coomassie gel below shows GST-tagged proteins used in pulldown.

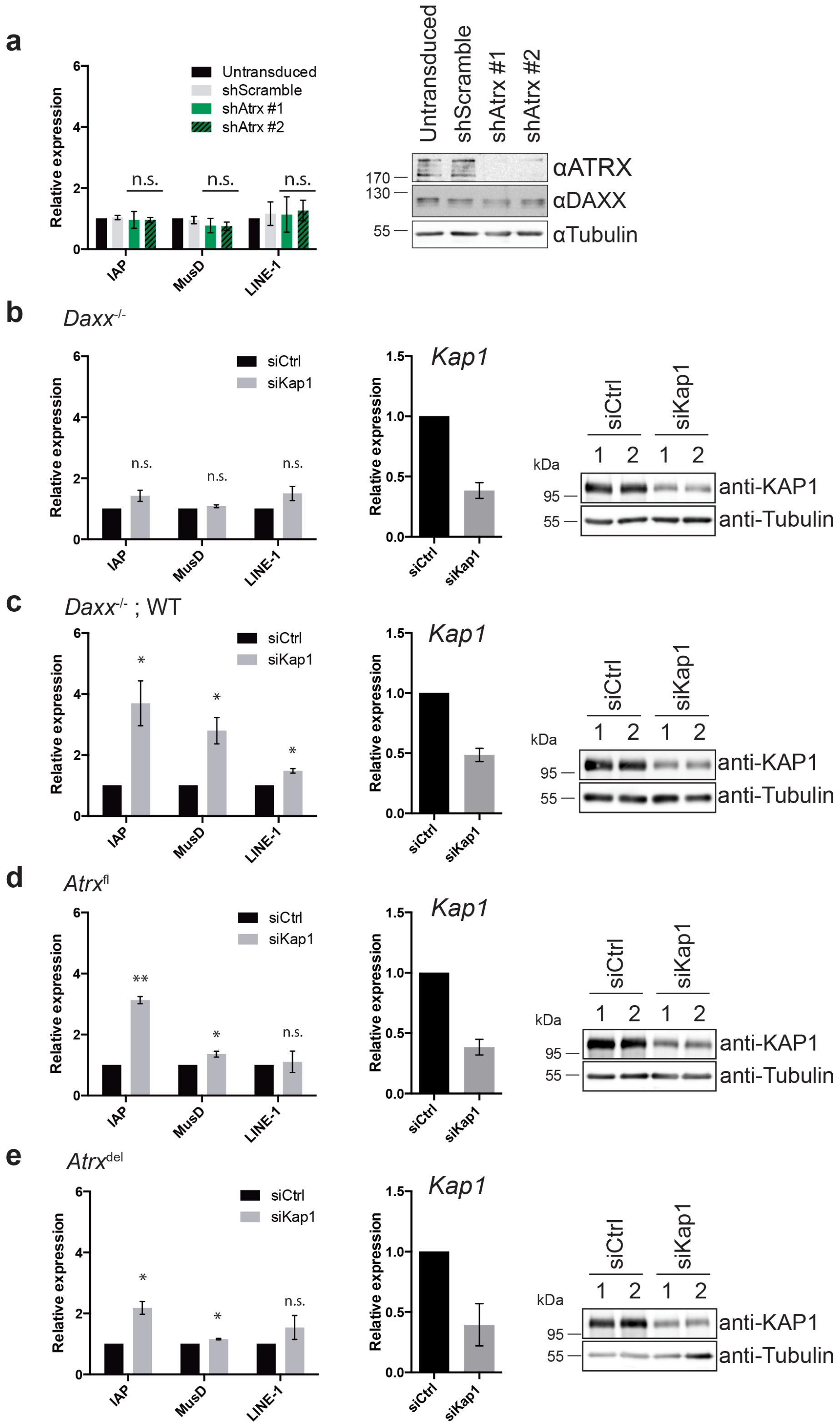

Supplementary Figure 3 | Epistatic relationship between KAP1 and DAXX, but not ATRX. (**a**) Expression of IAP, MusD, and LINE-1 elements as determined by RT-qPCR in untransduced C6 mESCs and cells transduced with control shRNA (shScramble), or shRNAs directed against *Atrx*. Data are shown as relative expression to *β-actin*. Shown are the results of two independent experiments (error bars represent S.E.M.). (**b-e**) Expression of IAP, MusD, and LINE-1 elements was determined via RT-qPCR in *Daxx*$^{-/-}$ (b), *Daxx*$^{-/-}$; WT (c), *Atrx*^{flox} (d), and *Atrx*^{del} mESCs (e) treated with either control siRNA (siCtrl) or siRNA directed against *Kap1* (siKap1). Data are shown as relative expression to *β-actin*. Shown are the results of three independent experiments (error bars represent S.E.M.). Respective mRNA and protein levels for Kap1 upon siRNA knockdown are shown adjacent to each panel (representing data of two experiments). Asterisks denote statistical significance as obtained by Student's *t*-test: *$p < 0.05$, **$p < 0.01$, n.s. = not significant.

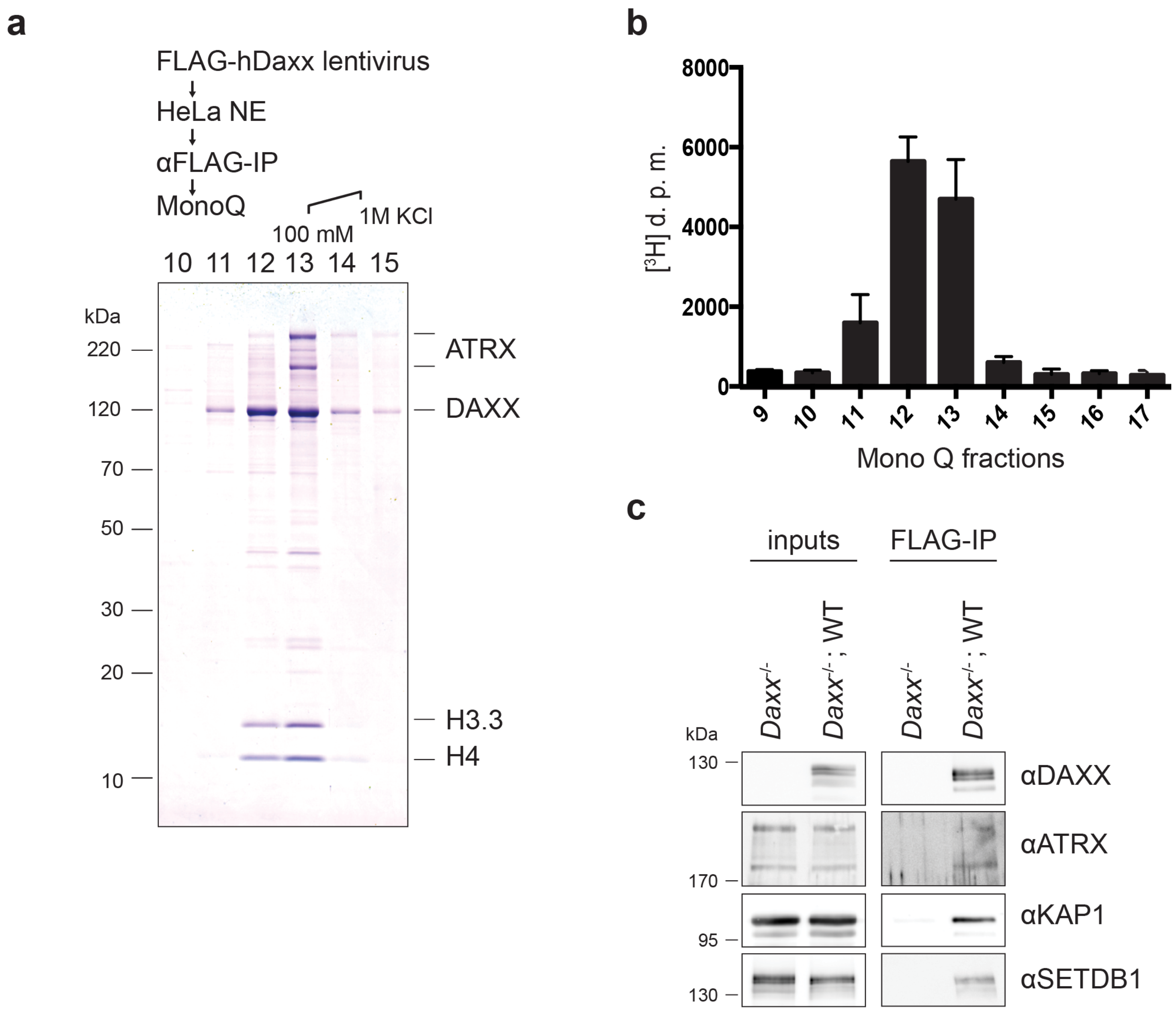

Supplementary Figure 4 | Methyltransferase activity on H3^{aa1-20} co-immunoprecipitates with FLAG-DAXX. (**a**) HeLa S3 nuclear extract expressing FLAG-hDAXX was subjected to anti-FLAG IP and subsequently fractionated via anion exchange chromatography (Mono Q). Coomassie stain shows Mono Q fractions. (**b**) Mono Q fractions were used in an *in vitro* histone methyltransferase assay using H3^{aa1-20} peptide as substrate in the presence of [^{3}H]-*S*-adenosyl methionine. Reactions were quantified via scintillation. Bar graphs depict the average of three technical replicates (error bars represent S.D.). (**c**) Immunoblots of inputs, anti-FLAG eluate from immunoprecipitation of FLAG-mDAXX in *Daxx*$^{-/-}$ mESCs, control (Daxx-/- mESCs with no transgene).

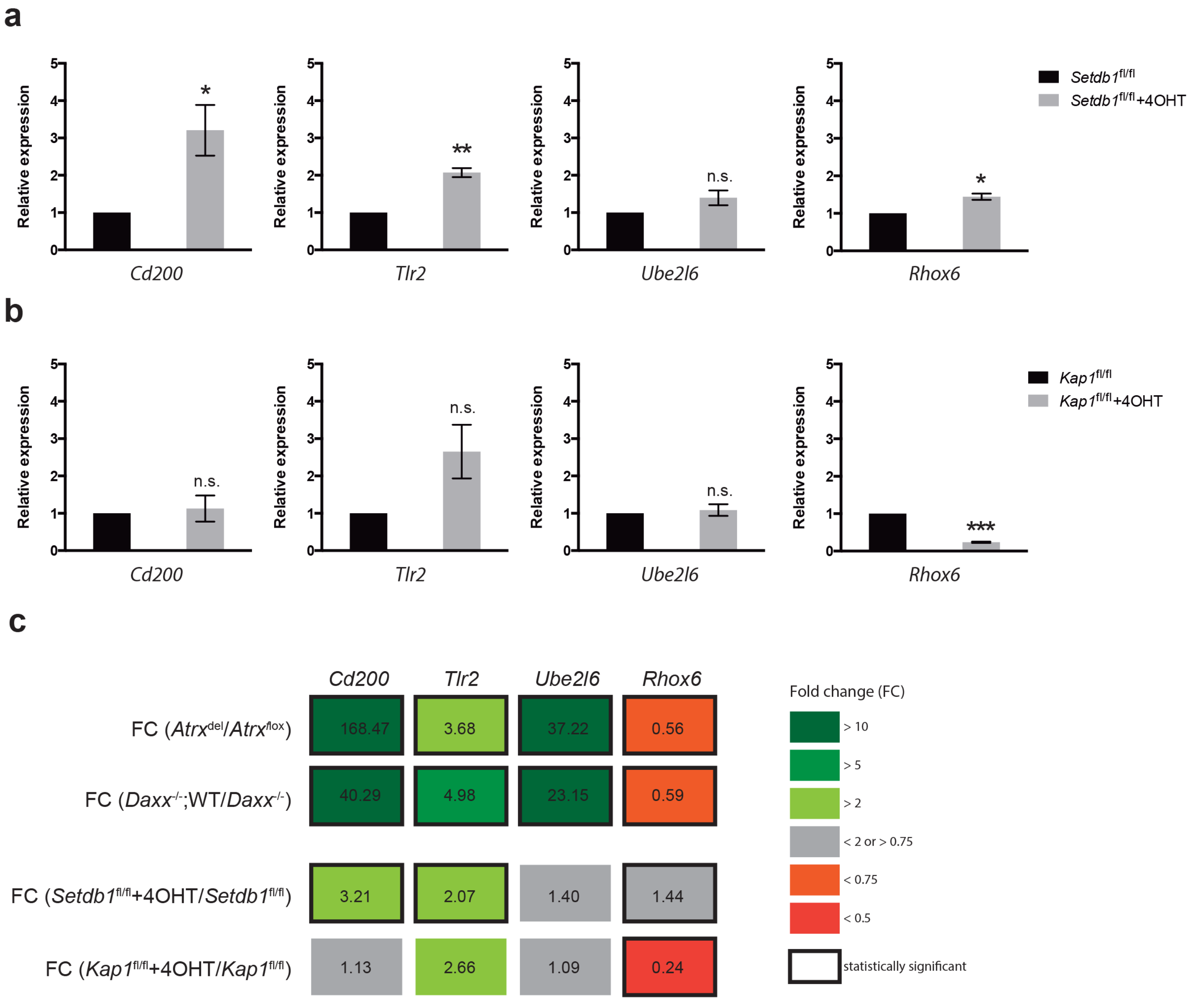

Supplementary Figure 5 | Expression of ATRX-DAXX-dependent genes in absence and presence of SETDB1 and KAP1. (a+b) Expression levels of *Cd200*, *Tlr2*, *Ube2l6*, and *Rhox6* as determined by RT-qPCR in (a) *Setdb1*$^{fl/fl}$ and 4OHT-treated *Setdb1*$^{fl/fl}$ mESCs, or in (b) *Kap1*$^{fl/fl}$ and *Kap1*$^{fl/fl}$ +4OHT mESCs. Data are presented as relative expression and were normalized to *β-actin*. Shown are the results of three independent experiments (error bars represent S.E.M.). Asterisks denote statistical significance as obtained by Student's *t*-test: *$p < 0.05$, **$p < 0.01$, ***$p < 0.001$, n.s. = not significant. (**c**) Summarized expression profiles for *Cd200*, *Tlr2*, *Ube2l6*, and *Rhox6* in presence and absence of ATRX, DAXX, SETDB1, and KAP1. Fold changes (absent/present) are listed and grouped by color. Statistical significance of observed fold change is indicated by black box frame.

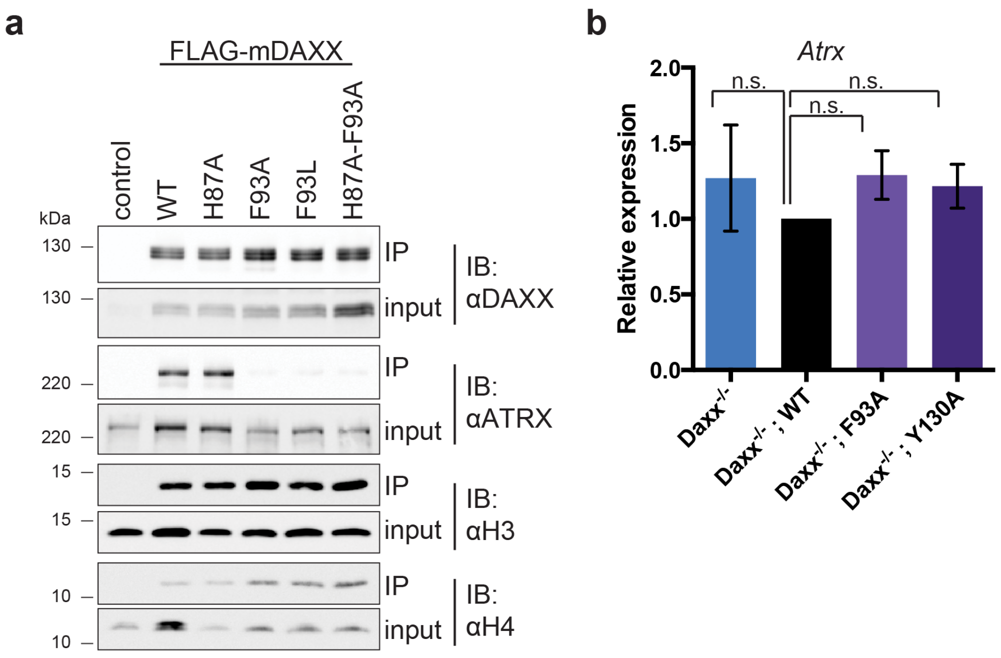

Supplementary Figure 6 | Additional biochemical and tumor mutant data with regard to the DAXX-ATRX interaction surface. (**a**) Immunoblots of FLAG-tagged mDAXX (WT, H87A, F93A, F93L, and F93A-H87A) immunoprecipitations and control (no FLAG-tagged transgene) in MEFs. Inputs and IP elutions were immunoblotted against anti-DAXX, anti-ATRX, anti-histone H3, and anti-histone H4. (**b**) Levels of *Atrx* mRNA in $Daxx^{-/-}$ mESCs and cells with indicated *Daxx* transgenes. Data are presented as relative expression and were normalized to *β-actin*. Shown are the results of two independent experiments (error bars represent S.E.M.). n.s. = not significant (Student's *t*-test).

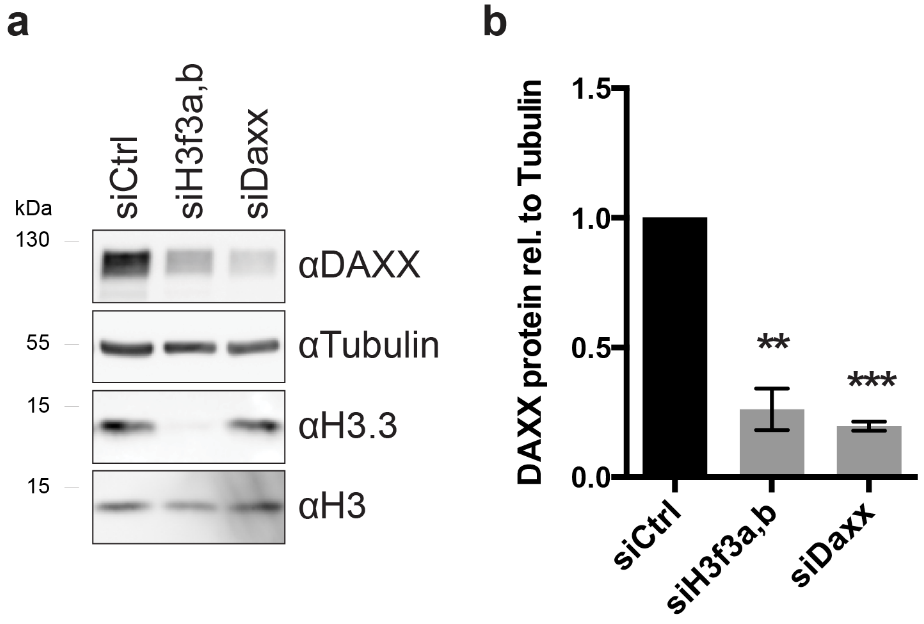

Supplementary Figure 7 | Knockdown of histone H3.3 in mESCs results in decreased DAXX protein levels. (**a**) C6 (wildtype) mESCs were treated with a non-targeting siRNA (siCtrl) or with siRNAs directed against *H3f3a* and *H3f3b* combined, or against *Daxx*. Immunoblots with anti-DAXX, anti-Tubulin, anti-H3.3, and anti-H3 are shown. (**b**) Bar graphs of anti-DAXX immunoblot in Fig. S7a band intensities quantified using Image J and normalized to anti-Tubulin signal. A total of three independent experiments were included in the quantification (error bars represent S.E.M.). Asterisks in denote significance by Student's *t*-test: $^{**}p < 0.01$, $^{***}p < 0.001$.

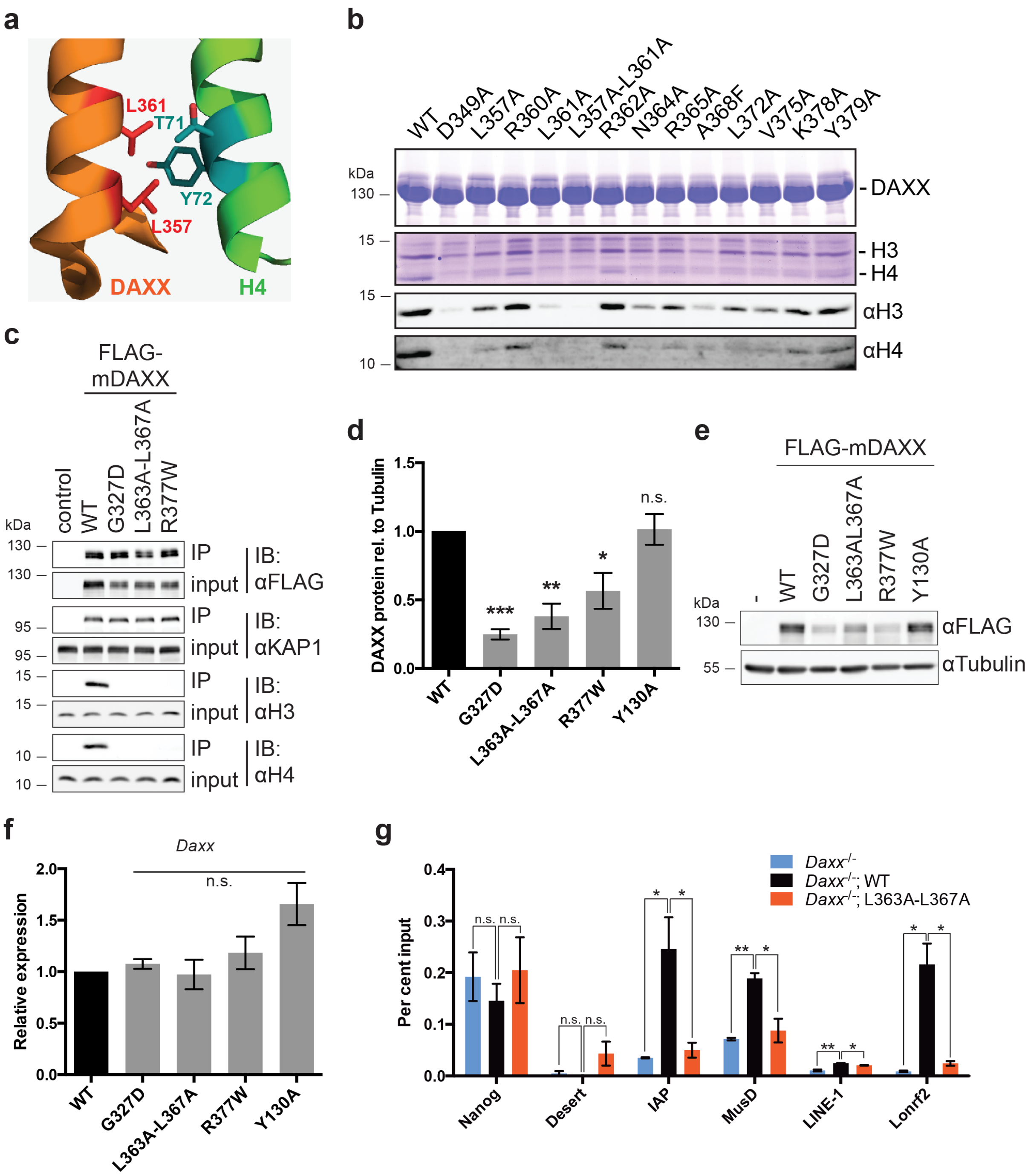

Supplementary Figure 8 | Synthesis and analysis of histone binding-deficient DAXX mutants. (**a**) Illustration of amino acid positions L363 and L367 in DAXX in the co-crystal structure with H3.3 and H4 (PDB ID: 4H9N). (**b**) FLAG-tagged DAXX WT and indicated mutants were expressed in Sf9 cells via a baculovirus expression system. Immunoprecipitation was performed using anti-FLAG M2 beads and proteins were separated by SDS-PAGE. Coomassie stained gel of immunoprecipitated DAXX and co-immunoprecipitated histones. Additionally, co-immunoprecipitated histones are identified via immunoblot with anti-H3 and anti-H4 antibodies. (**c**) Co-immunoprecipitation of FLAG-tagged mDAXX (WT, G327D, L363A-L367A, R377W) alongside control (no FLAG-tagged transgene) in MEFs. Inputs and IP elutions were immunoblotted against anti-FLAG, anti-KAP1, anti-H3, and anti-H4. (**d**) Bar graphs of anti-DAXX immunoblot in Fig. 4b band intensities quantified using Image J and normalized to anti-Tubulin signal. A total of four independent experiments were included in the quantification (error bars represent S.E.M.). (**e**) C6 (wildtype) mESCs were transduced with the indicated FLAG-tagged *Daxx* transgenes. Immunoblots for anti-FLAG and anti-Tubulin are shown. (**f**) Levels of *Daxx* mRNA in *Daxx*$^{-/-}$ mESCs stably transduced with the indicated *Daxx* transgenes. Data are presented as relative expression and were normalized to *β-actin*. Shown are the results of two independent experiments (error bars represent S.E.M.). (**g**) Anti-HA ChIP-qPCR for indicated genomic regions in mESCs with stable H3.3-HA knock-in. Shown are results from *Daxx*$^{-/-}$ mESCs, and rescue of these cells with *Daxx* transgenes (WT, L363A-L367A). Data are presented as per cent input (error bars depict S.E.M.). Shown are the results of two independent experiments. Graphs for *Daxx*$^{-/-}$ and *Daxx*$^{-/-}$; WT are taken from the same experiment as in Fig. 2g. Asterisks in (d,f,g) denote significance by Student's *t*-test: *$p < 0.05$, **$p < 0.01$, ***$p < 0.001$, n.s. = not significant.

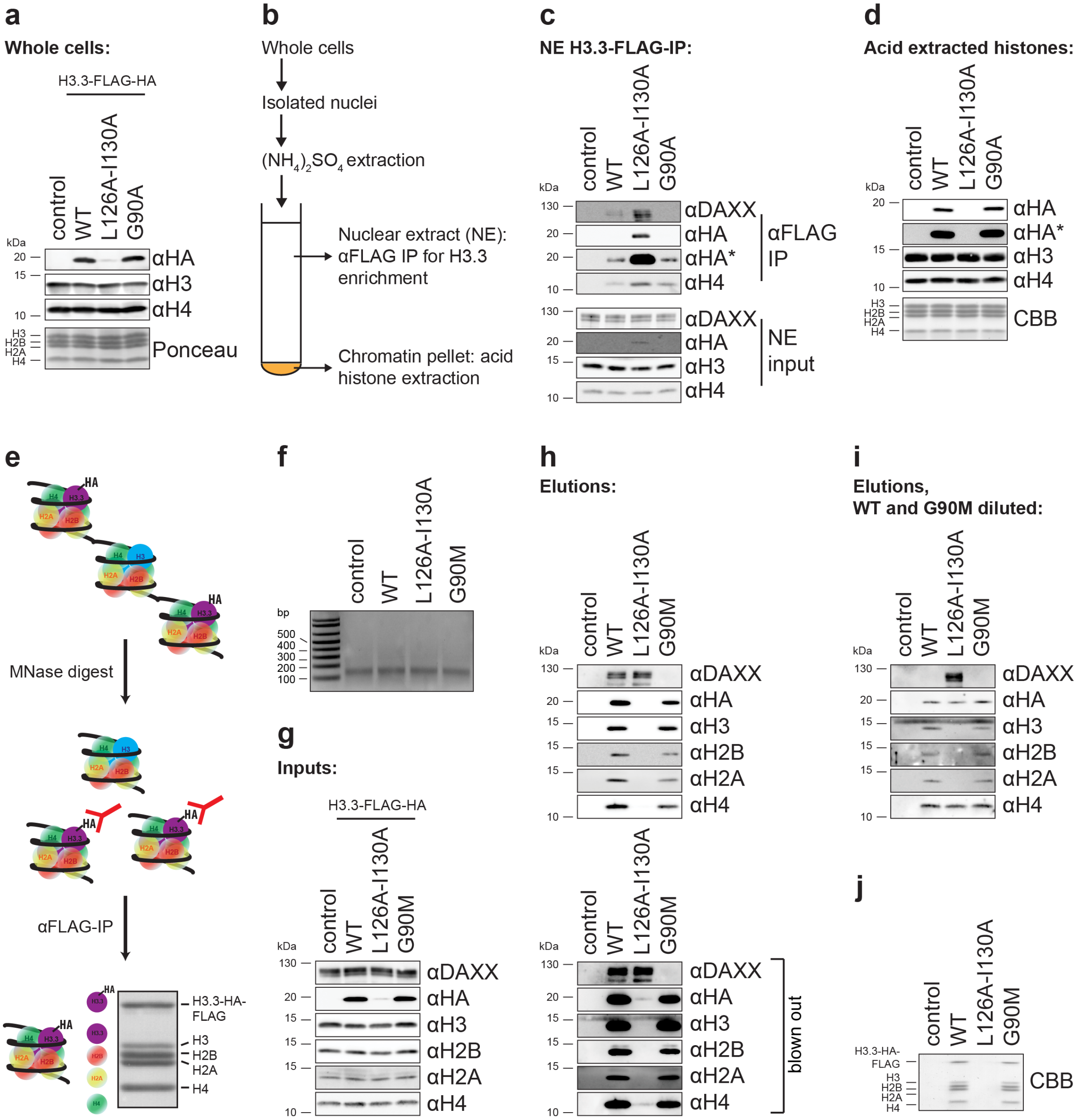

Supplementary Figure 9 | Histone L126A-I130A mutant interacts with DAXX but does not incorporate into chromatin. (**a**) Immunoblots against HA, Histones H3 and H4 in whole-cell lysates of untranduced or transduced MEFs with different H3.3-HA-FLAG transgenes. Ponceau stain shows histones present in the sample. (**b**) Nuclei were isolated from whole cell MEFs and subjected to ammonium sulfate extraction (0.4M). Nuclear contents were separated into a chromatin pellet and a soluble nuclear extract via ultracentrifugation (100K x G). The soluble fraction was subjected to anti-FLAG IP to enrich for H3.3-HA-FLAG-tagged pre-deposition histones, while the chromatin pellet was subjected to acid histone extraction. (**c**) Immunoblots on inputs and anti-FLAG immunoprecipitation of samples from the soluble nuclear extract from cell lines with the noted transgenes. (**d**) Immunoblots on acid extracted histones were performed against HA and histones H3 and H4. Coomassie stained gel shows loading for extracted histones. Images in (c) and (d) marked with an asterisk denote a duplicated, blown-out version of the respective anti-HA blot. (**e**) MEF lysates were subjected to digestion with MNase to generate mononucleosomes. H3.3-HA-FLAG containing nucleosomes and pre-deposition complexes were enriched via anti-FLAG immunoprecipitation. The constituents of immunoprecipitated heterotypic nucleosomes are labeled on a representative Coomassie-stained gel. (**f**) The agarose gel shows DNA of mononucleosomes generated from lysates of MEFs expressing the indicated H3.3-HA-FLAG-tagged transgenes. (**g**) Immunoblots of inputs for the anti-FLAG immunoprecipitation eluate shown in (h). (**h**) Immunoblots of eluate from Anti-FLAG immunoprecipitation of mononucleosomes generated from the noted cells lines. The lower panel shows overexposed images of the immunoblot images above. (**i**) Immunoblot of anti-FLAG immunoprecipitations from noted transgenic celllines. Here the eluate for histone H3.3 WT and G90M were diluted 20-fold. (**j**) Coomassie stained gel shows the presence of heterotypic nucleosomes within the elutions for H3.3 WT and G90M.

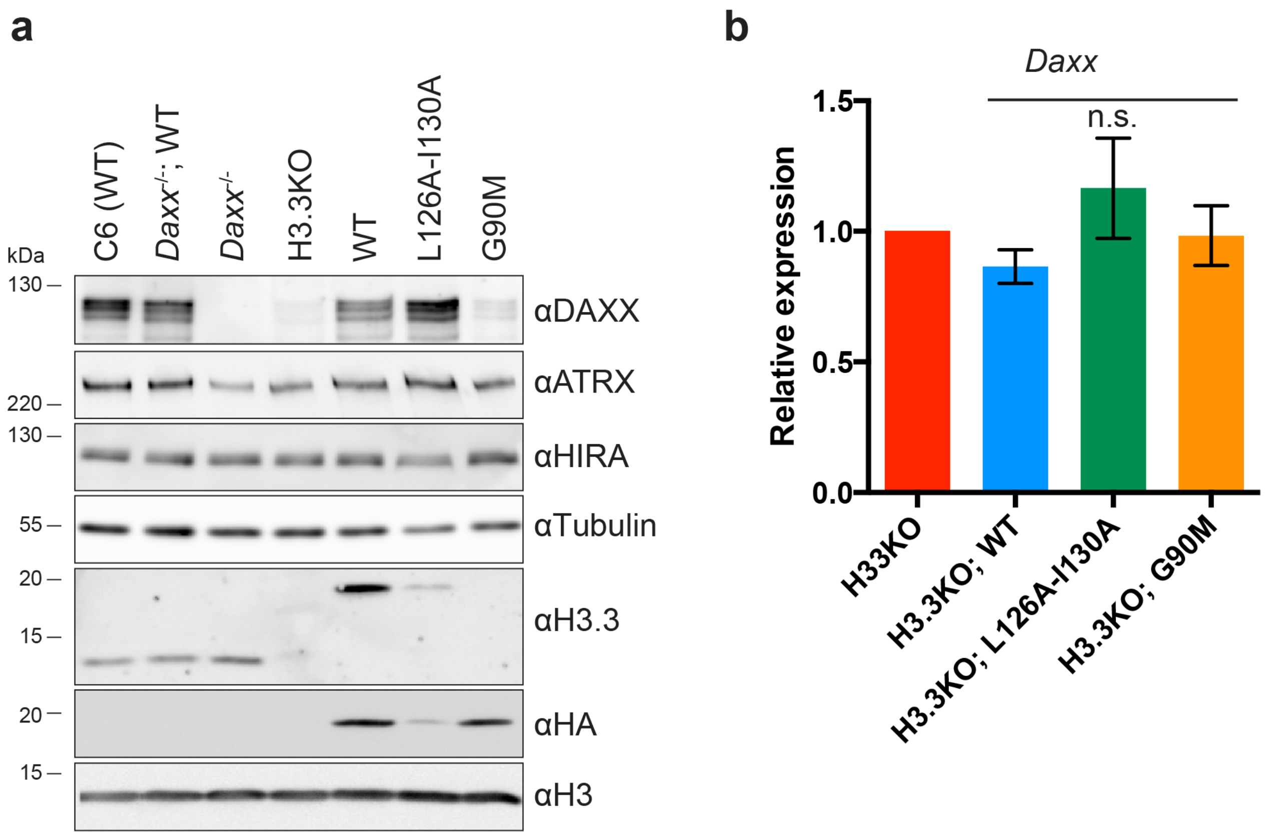

Supplementary Figure 10 | DAXX protein and mRNA levels in H3.3KO mESCs rescued with different H3.3 transgenes. (**a**) Immunoblots of whole cell extract from indicated cell lines for the split panels shown in Figure 4a and e. (**b**) RT-qPCR showing levels of *Daxx* mRNA in H3.3 KO mESCs stably transduced with the indicated H3.3 transgenes. Data are presented as relative expression and were normalized to *β-actin*. Shown are the results of four independent experiments (error bars represent S.E.M.). n.s. = not significant (Student's *t*-test).

Figure 1

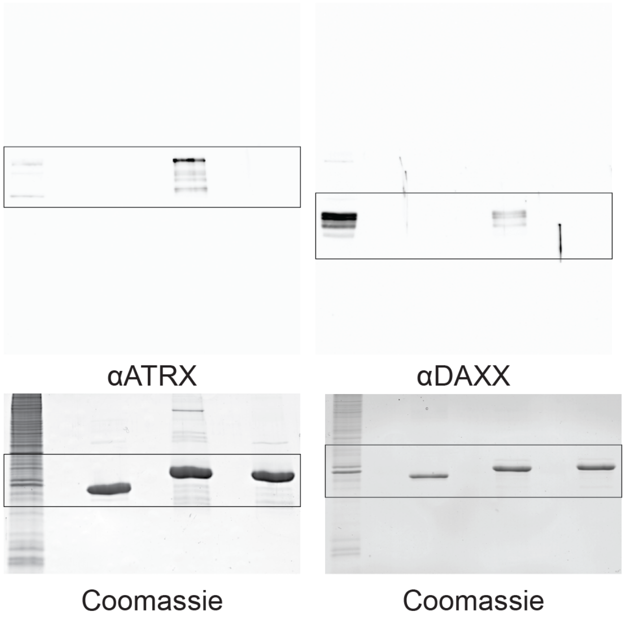

Figure 2

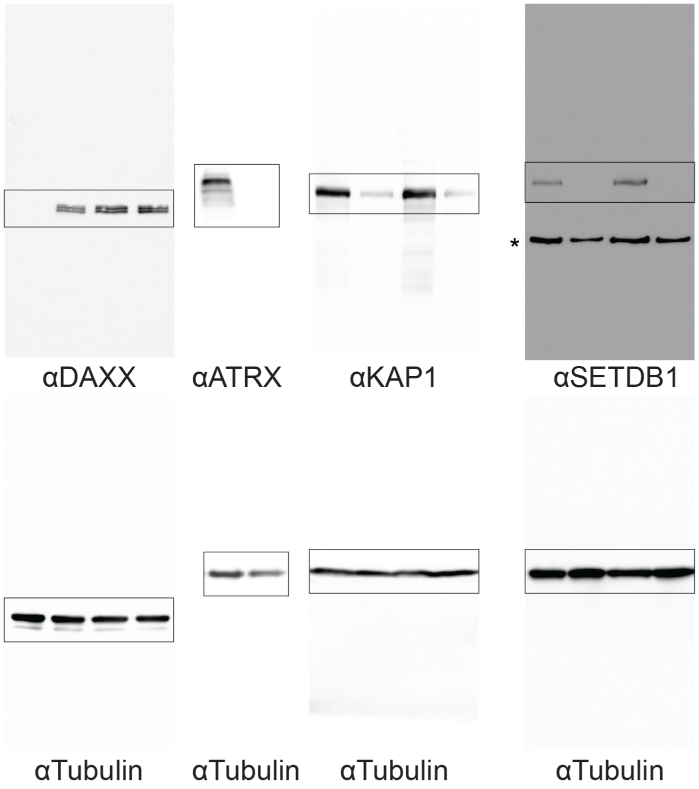

Figure 3

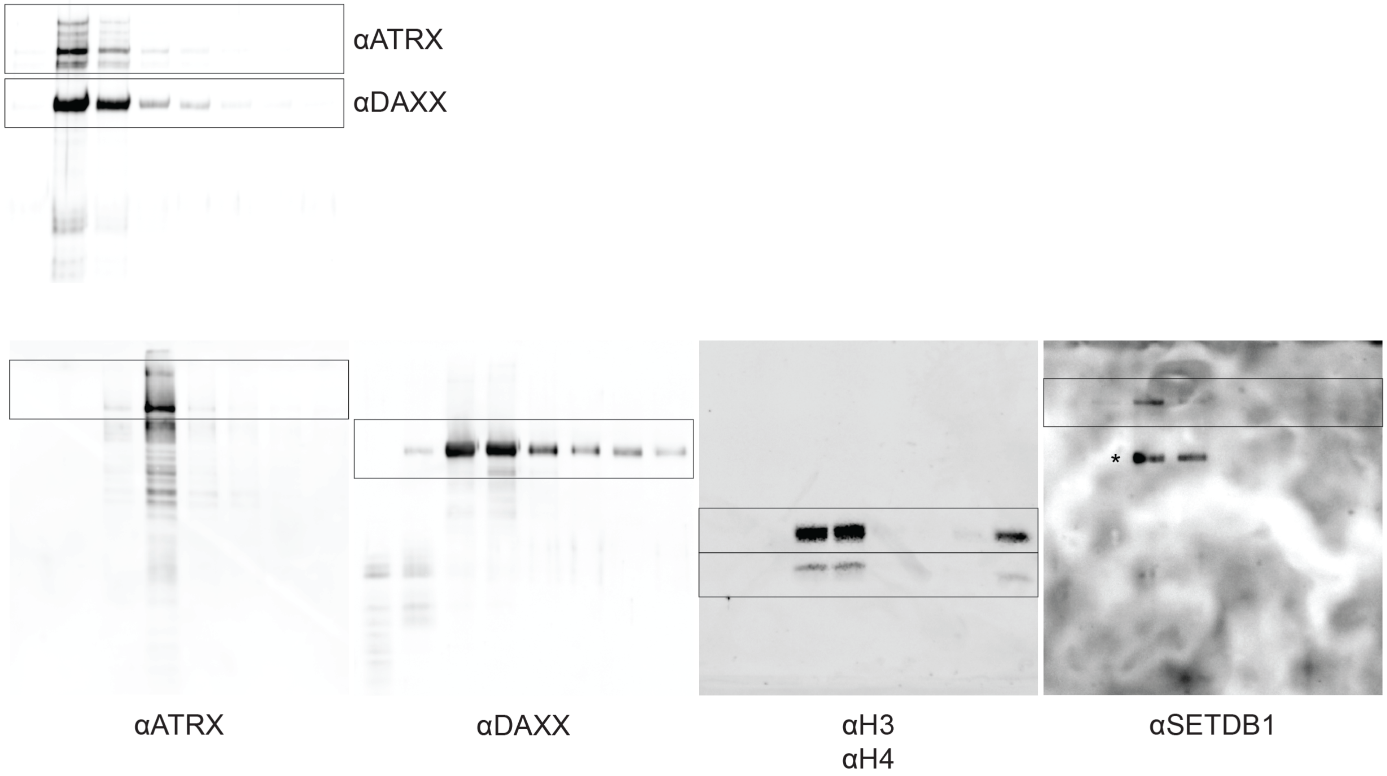

*: unspecific band seen with Abcam anti-SETDB1 (ab5430) antibody (size 120 kDa; SETDB1 is 150 kDa)

Figure 3, continued

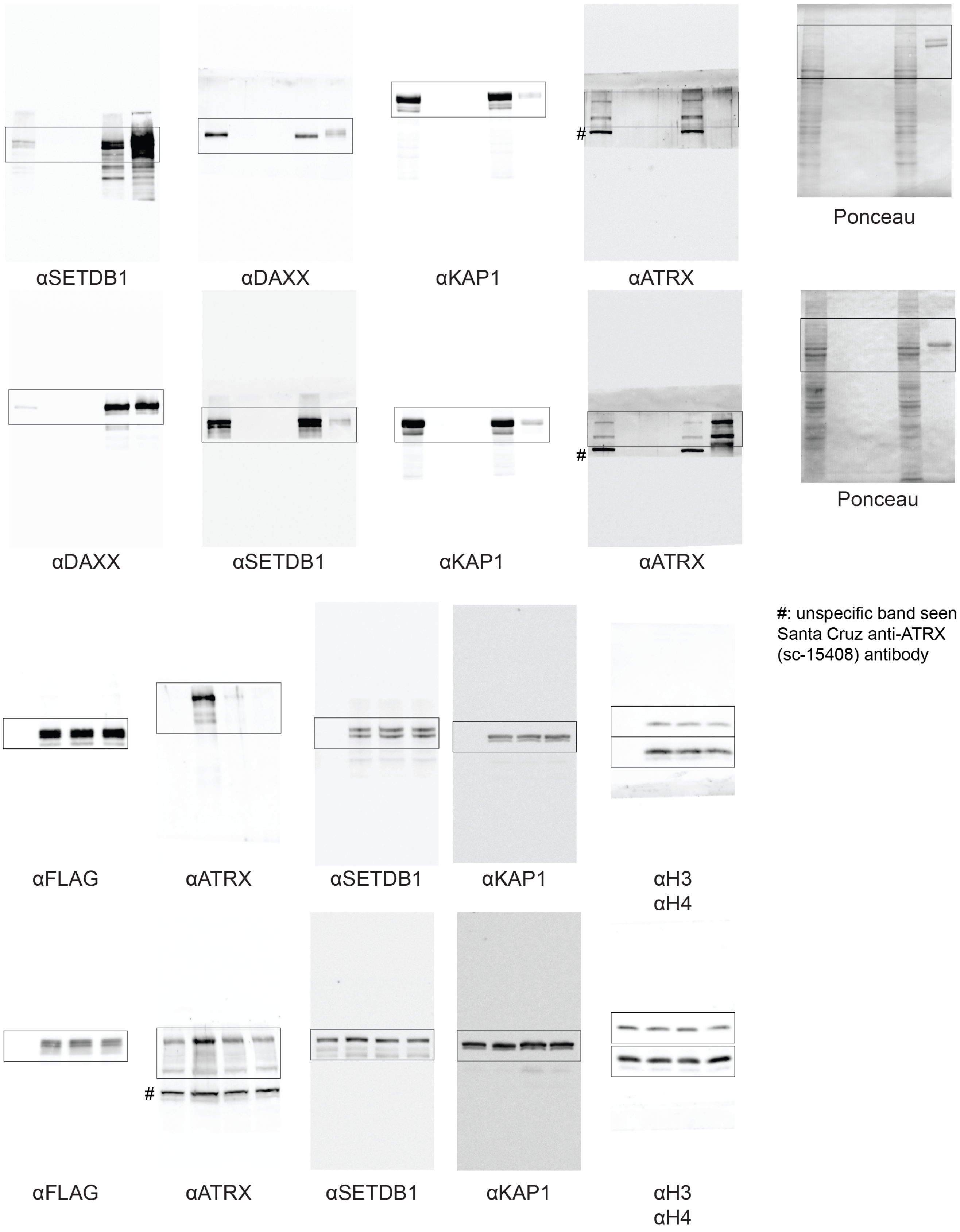

#: unspecific band seen with Santa Cruz anti-ATRX (sc-15408) antibody

Figure 4

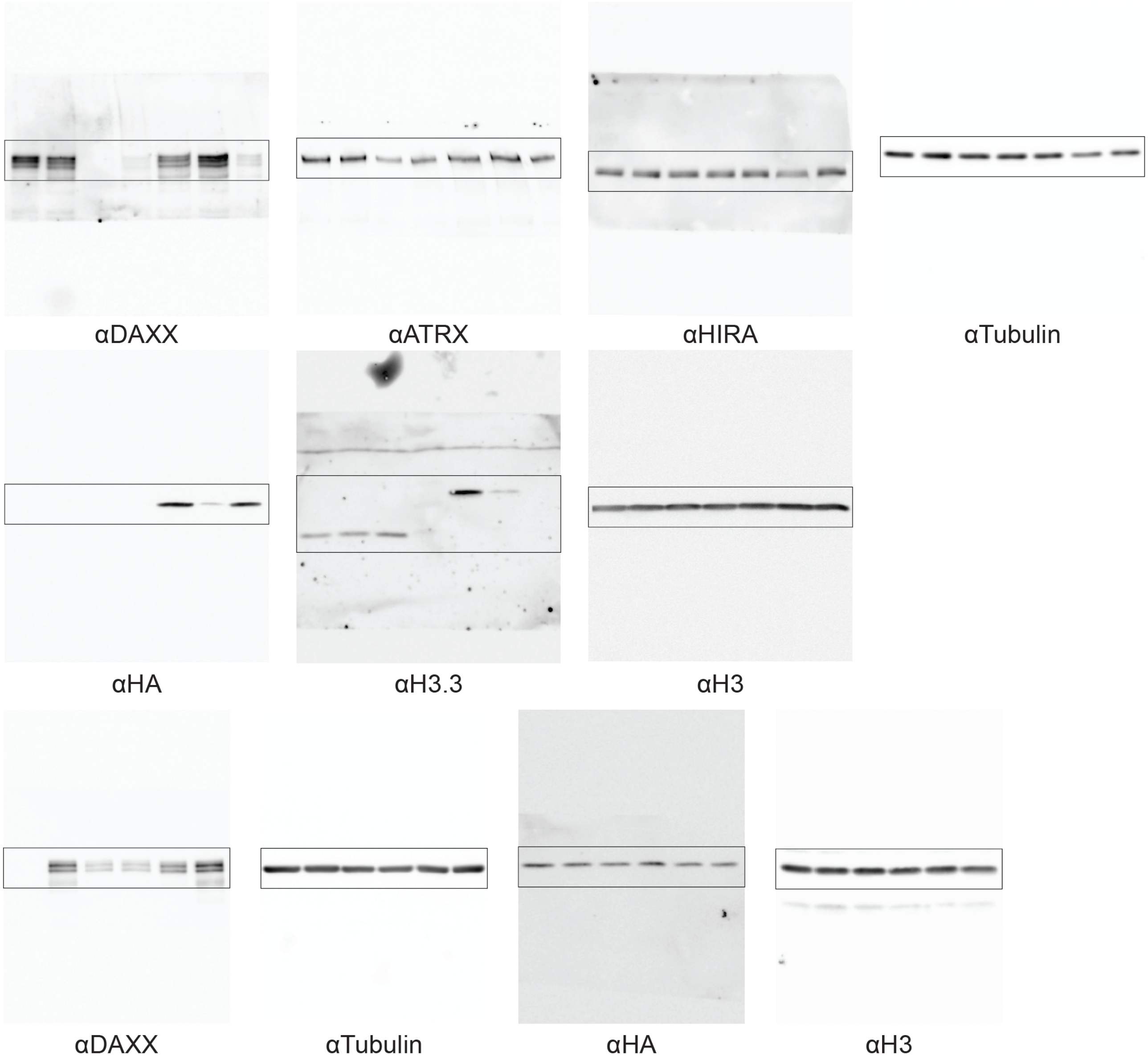

Figure 6

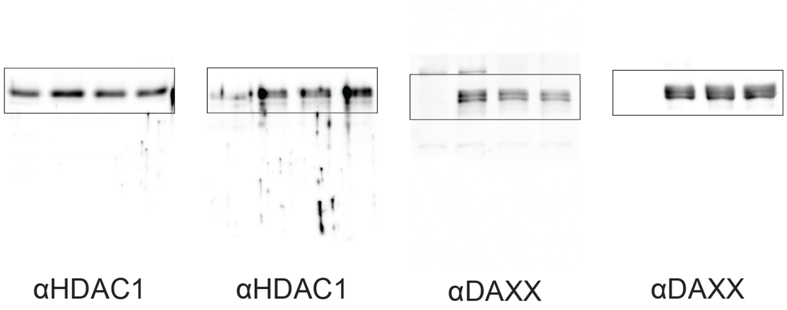

Figure 6, continued

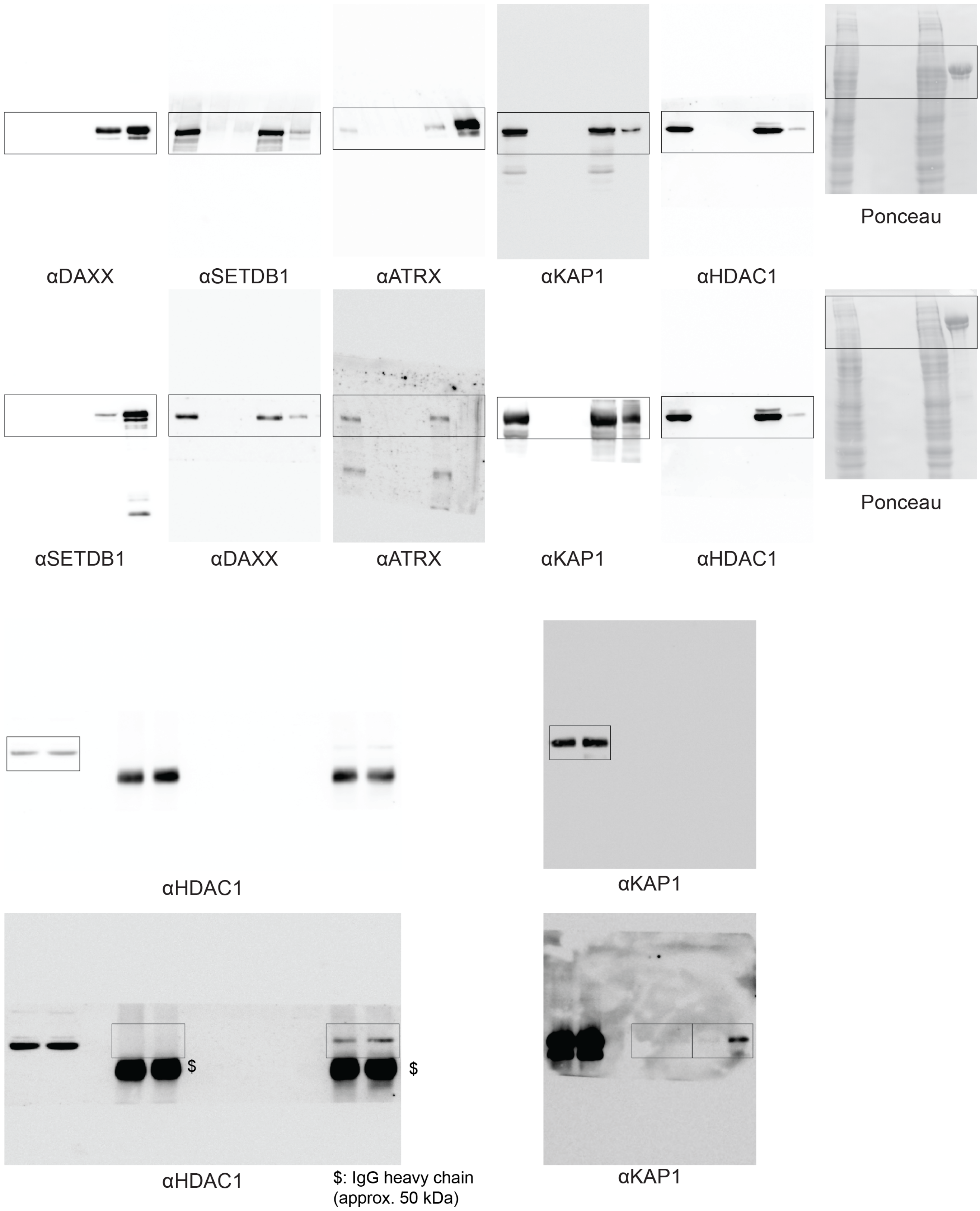

Figure S2

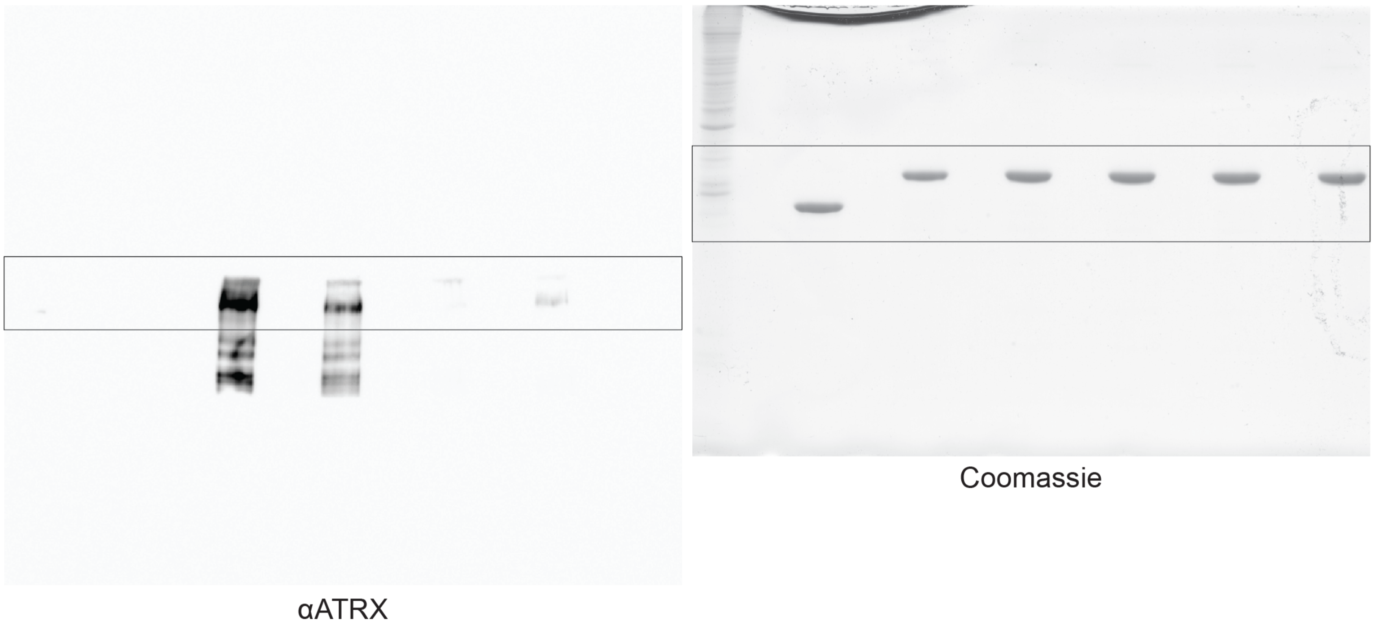

Figure S3

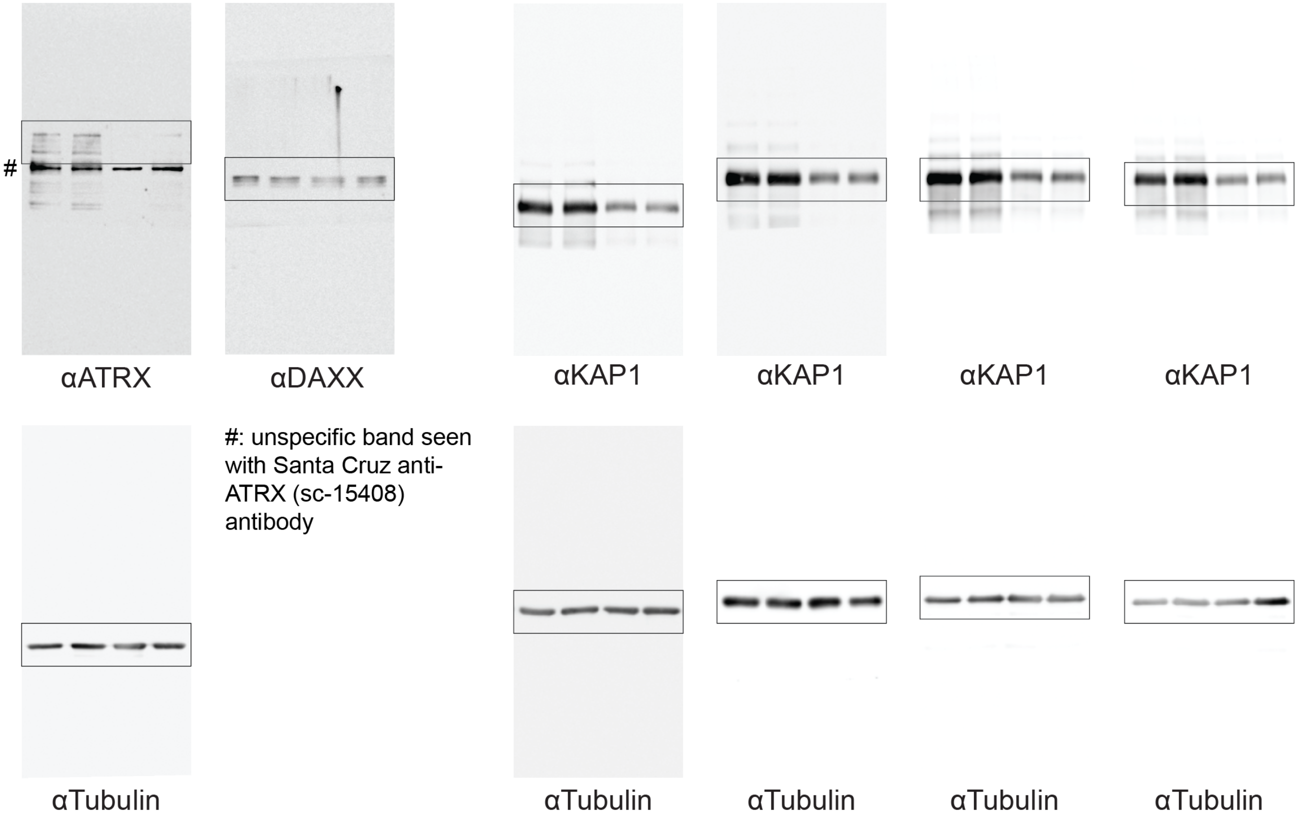

Figure S4

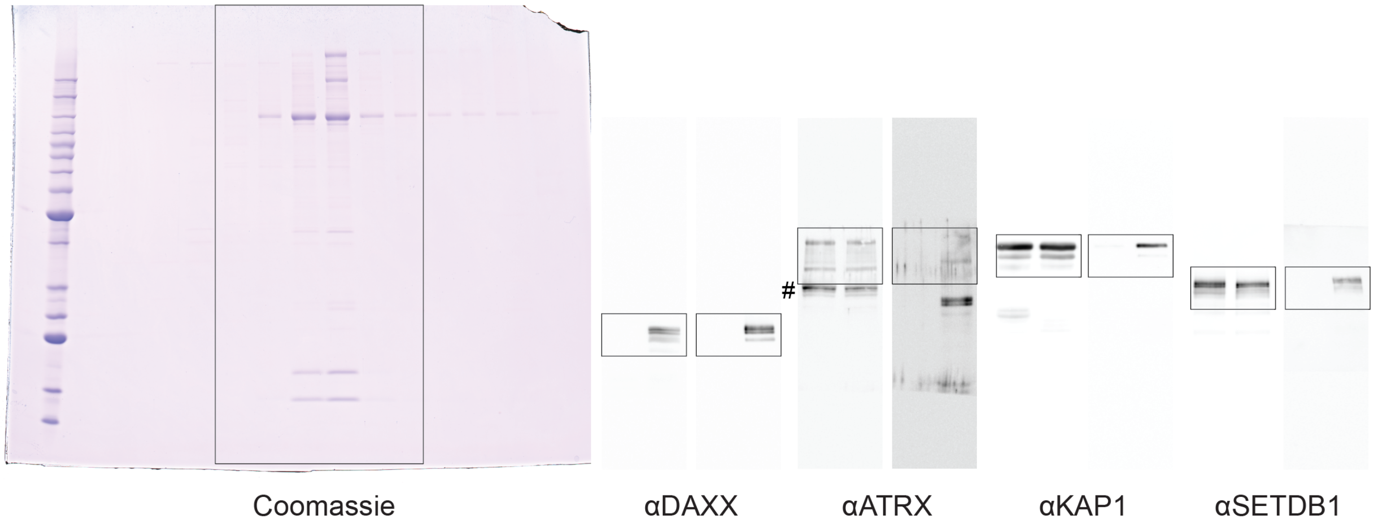

Figure S6

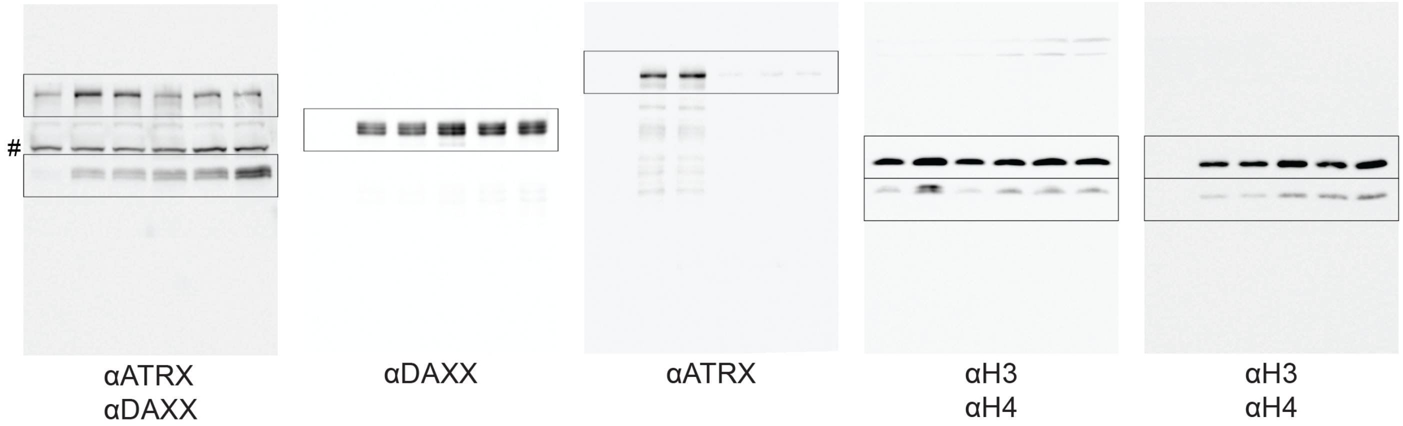

#: unspecific band seen with Santa Cruz anti-ATRX (sc-15408) antibody

Figure S7

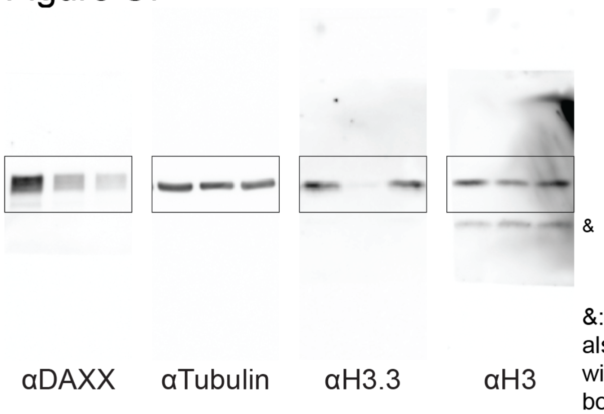

&: membrane had also been probed with anti-H4 anti-body

Figure S8

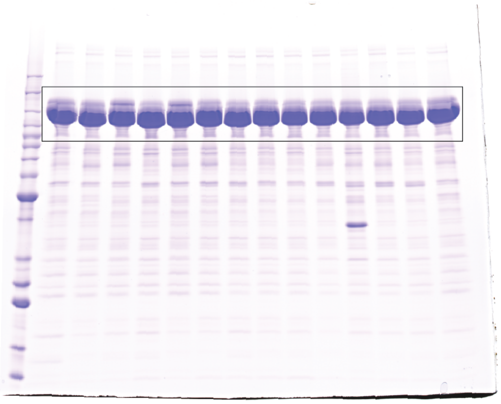

Coomassie

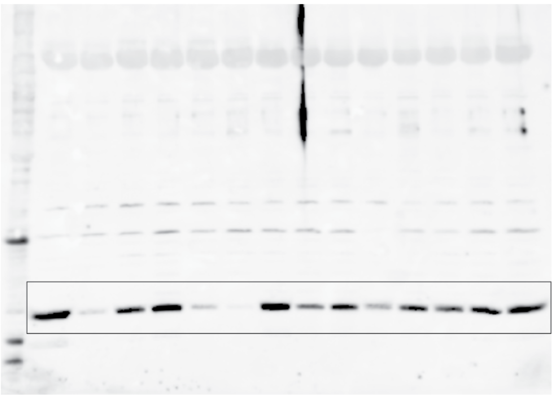

αH3

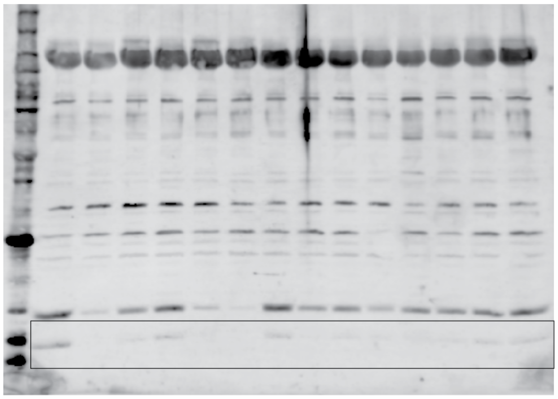

αH4

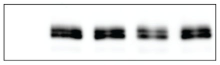

αFLAG

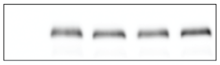

αKAP1

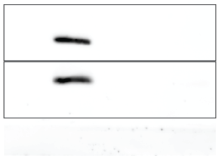

αH3
αH4

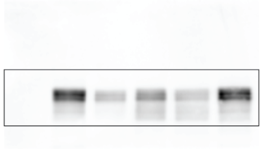

αFLAG

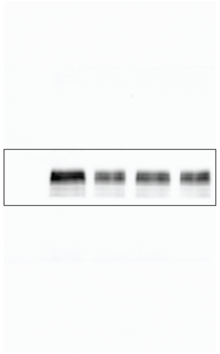

αFLAG

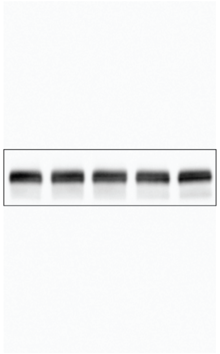

αKAP1

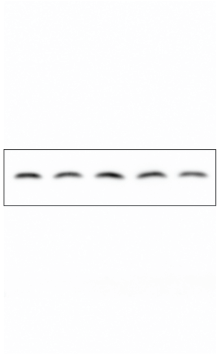

αH3

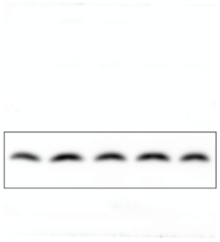

αH4

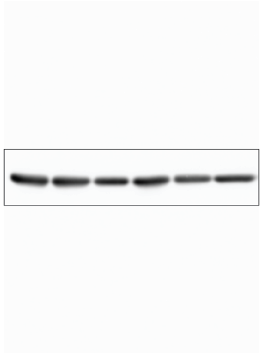

αTubulin

Figure S9

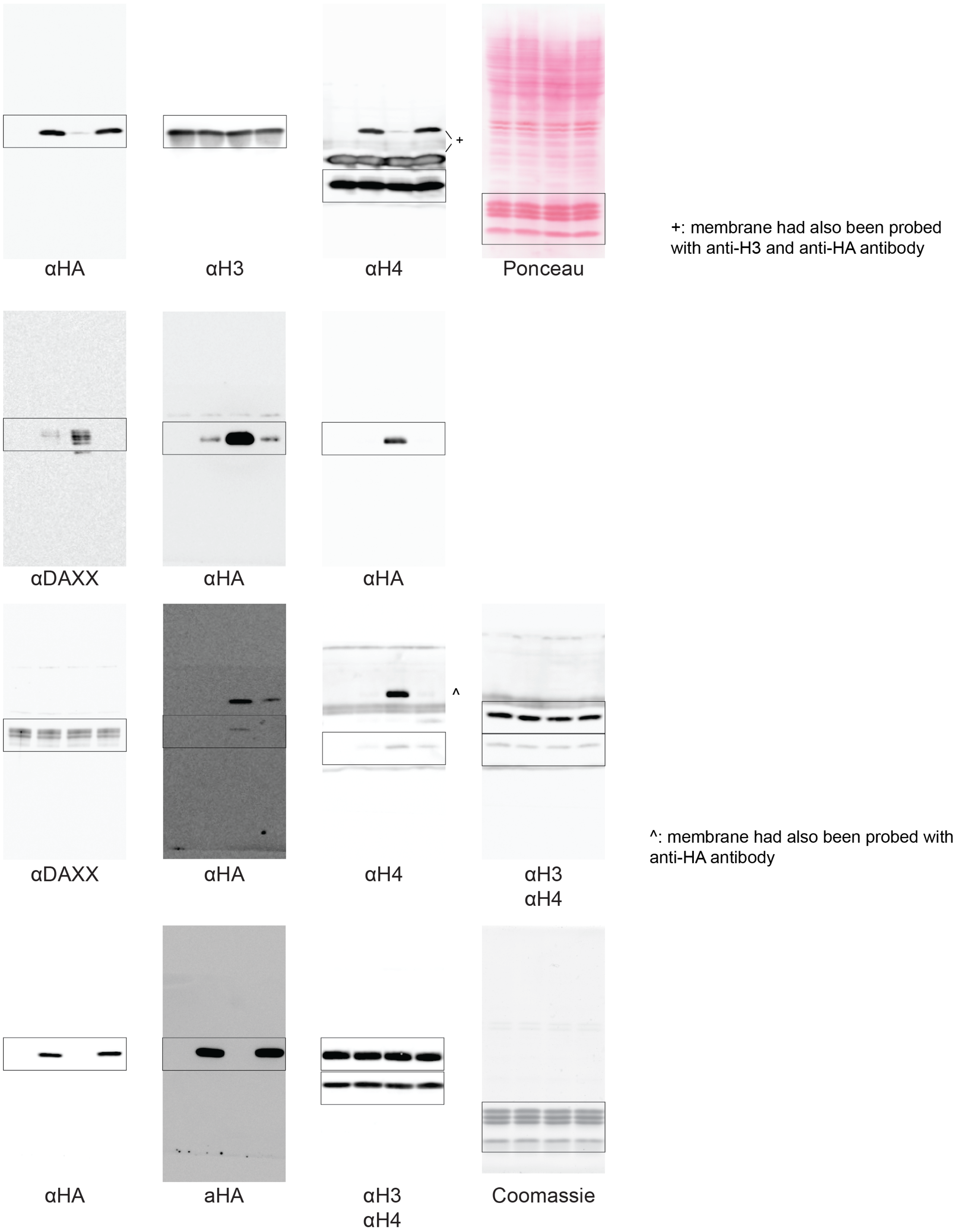
αHA
αH3
αH4
+
Ponceau
+: membrane had also been probed with anti-H3 and anti-HA antibody
αDAXX
αHA
αHA
αDAXX
αHA
αH4
^
αH3
αH4
^: membrane had also been probed with anti-HA antibody
αHA
aHA
αH3
αH4
Coomassie

Figure S9, continued

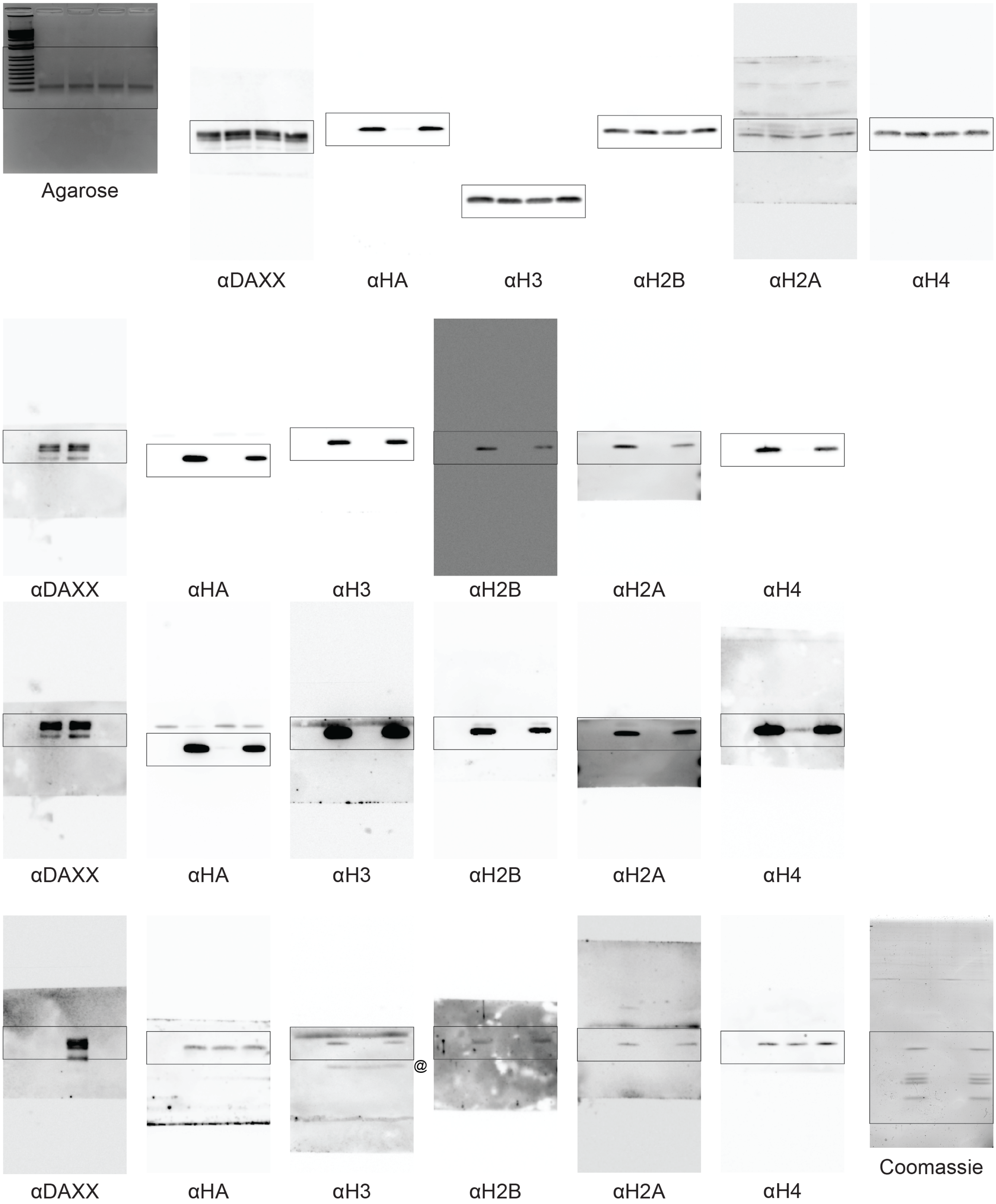

@: membrane had also been probed with anti-H4 antibody

Supplementary Figure 11 | Uncropped Western blot and gel images. Cropped regions used in the main and supplementary figures are outlined.

Supplementary Table 1 | Crystallographic data and refinement statistics.

	DAXX 4HB – ATRX DBM Complex (5Y6O)
**Data collection**	
Space group	$P3_1$ 2 1
Cell dimensions	
a, *b*, *c* (Å)	78.7, 78.7, 503.2
α, β, γ (°)	90, 90, 120
Resolution (Å)	50-3.10 (3.27-3.10)[a]
R_{pim} (%)	3.3 (52.7)
$I/\sigma I$	14.4 (1.1)
Completeness (%)	99.9 (99.9)
Redundancy	8.0 (6.9)
**Refinement**	
No. reflections (unique)	34,234
$R_{work/}$ R_{free} (%)	22.4/29.7
No. atoms	
Protein	7,467
B-factors	
Protein	72.9
R.m.s deviations	
Bond lengths (Å)	0.01
Bond angles (º)	1.43

[a] Highest resolution shell is shown in parenthesis. One crystal was used for the data.

	DAXX WT		H3.3 WT		H3.3 L126A-I130A	
	FC (linear)	FC (log_2)	FC (linear)	FC (log_2)	FC (linear)	FC (log_2)
Samd9l	23.54869865	4.557575431	6.874282897	2.781209224	6.638212462	2.730794805
Naprt1	2.412861095	1.270744864	2.555906385	1.353834996	2.285638167	1.192597033
Specc1	0.469725324	-1.09011072	0.36817246	-1.44154638	0.360781924	-1.470801037
Smoc1	0.449863531	-1.152440678	0.347409709	-1.525290022	0.26264883	-1.928792937
Stra8	0.426523862	-1.229301639	0.267159207	-1.904228358	0.033840032	-4.885125257
Pde3b	0.417611884	-1.259765328	0.393648443	-1.345020323	0.380865578	-1.392646189
Slc43a3	0.352716466	-1.503419168	0.328328349	-1.606788772	0.19284913	-2.374455457
Sohlh2	0.329988764	-1.599511193	0.294575152	-1.763292354	0.303325983	-1.72105901
Arhgap44	0.328468205	-1.606174367	0.403545766	-1.309195797	0.275743186	-1.85860286
Rnf128	0.325927984	-1.617374868	0.341297264	-1.550899246	0.190466934	-2.392387534
Rbm20	0.308252045	-1.697817629	0.359024112	-1.477847356	0.473359074	-1.078993117
Zcchc12	0.285465612	-1.80861113	0.265114243	-1.915313915	0.203119719	-2.299597791
Med12l	0.273064948	-1.872683961	0.404347819	-1.306331265	0.26226571	-1.930898901
Ushbp1	0.245637874	-2.025395073	0.258180434	-1.953548424	0.232650379	-2.103764558
Rbm44	0.227034054	-2.139019384	0.440049748	-1.184261464	0.284416474	-1.813923064
Rhobtb1	0.201745124	-2.30939429	0.210025843	-2.251361237	0.095755186	-3.384505566
Tspan2	0.194752685	-2.360284876	0.208226267	-2.263776024	0.492309737	-1.022361821
Timp2	0.191169273	-2.38707744	0.327482259	-1.610511343	0.318226987	-1.651871907
Popdc3	0.150424162	-2.732891775	0.400518176	-1.32006038	0.227975087	-2.133051919
4930524B15Rik	0.093282743	-3.422245978	0.173632754	-2.525888972	0.143249993	-2.803393026
Brdt	0.086600691	-3.529477653	0.330779141	-1.596059834	0.153148263	-2.706999091
Heph	0.084570787	-3.563696786	0.220812199	-2.179108217	0.05054877	-4.306180202
Serpina3k	0.007382353	-7.08170356	0.083693378	-3.578742712	0.04695824	-4.412477853
Serpina3m	0.000761207	-10.35942355	0.101847379	-3.295519241	0.066137517	-3.918387306

Supplementary Table 2 | Fold changes (linear and log2) for genes differentially expressed in the same direction within the DAXX WT and H3.3 WT/L126A-I130A overlap. Genes up-regulated by DAXX are shown in green, genes down-regulated by DAXX in red. Genes highlighted in yellow were subjected to validation via qRT-PCR (see Fig. 5c,d).

Supplementary Table 3 | List of antibodies.

Antibody [clone]	Company (Cat. No.)	Source	Application	Dilution
anti-ATRX [H-300]	Santa Cruz Biotechnology (sc-15408)	rabbit polyclonal	Immunoblot	1:250 - 1:500
anti-DAXX [M-112]	Santa Cruz Biotechnology (sc-7152)	rabbit polyclonal	Immunoblot	1:500 - 1:2,000
anti-DDK/FLAG tag	Origene (TA100023)	rabbit polyclonal	Immunoblot	1:1,000 - 1:2,000
anti-KAP1 [2OC1]	Abcam (ab22553)	mouse monoclonal	Immunoblot	1:2,000
anti-SETDB1 [5H6A12]	Abcam (ab107225)	mouse monoclonal	Immunoblot	1:500 - 1:1,000
anti-SETDB1	Abcam (ab5430)	rabbit polyclonal	Immunoblot	1:500 - 1:1,000
anti-beta-Tubulin [2G7D4]	Genscript (A01717-40)	mouse monoclonal	Immunoblot	1:10,000 - 1:50,000
anti-H3 general	in house	rabbit polyclonal	Immunoblot	1:2,000 - 1:10,000
anti-H4 general	in house	rabbit polyclonal	Immunoblot	1:500 - 1:1,000
anti-H3.3	Millipore (09-838)	rabbit polyclonal	Immunoblot	1:500
anti-HA [12CA5]	in house	mouse monoclonal	Immunoblot<br>ChIP	1:1,000-1:2,000<br>4 µg per sample
anti-HDAC1	Millipore (06-720)	rabbit polyclonal	Immunoblot	1:1000
anti-HDAC1	Novus Biologicals (NB500-124)	rabbit polyclonal	Immunoblot<br>IP	1:2000<br>4 µg per sample
anti-H2A	in house	rabbit polyclonal	Immunoblot	1:500
anti-H2B	in house	rabbit polyclonal	Immunoblot	1:1000
anti-HIRA [WC15]	in house	mouse monoclonal	Immunoblot	1:500
Anti-Rabbit IgG (H + L)-HRP Conjugate	BIO-RAD (1706515)	goat polyclonal	Immunoblot	1:3000
Anti-Mouse IgG (H + L)-HRP Conjugate	BIO-RAD (1706516)	goat polyclonal	Immunoblot	1:3000

Supplementary Table 4 | List of primers.

Primer name	Sequence (5' - 3')	Application
mGapdh F	ATGAATACGGCTACAGCAACAGG	RT-qPCR
mGapdh R	CTCTTGCTCAGTGTCCTTGCTG	RT-qPCR
mb-actin ex 2 F	GAACCCTAAGGCCAACCGTG	RT-qPCR
mb-actin ex 3 R	GGAGTCCATCACAATGCCTG	RT-qPCR
IAP F	CGGGTCGCGGTAATAAAGGT	RT-qPCR
IAP R	ACTCTCGTTCCCCAGCTGAA	RT-qPCR
MusD F	GATTGGTGGAAGTTTAGCTAGCAT	RT-qPCR
MusD R	TAGCATTCTCATAAGCCAATTGCAT	RT-qPCR
LINE-1 F	TTTGGGACACAATGAAAGCA	RT-qPCR
LINE-1 R	CTGCCGTCTACTCCTCTTGG	RT-qPCR
mAtrx ex1 F	GTCCGAGCCAAAAACATGAC	RT-qPCR
mAtrx ex2 R	GTCATGAAGCTTCTGCACCA	RT-qPCR
mCd200 ex1 F	AGAGCAAGGATGGGCAGTCT	RT-qPCR
mCd200 ex2-3 R	GTCACCACTTCCACTTGAGC	RT-qPCR
mKap1 ex3 F	TGAACCACTTTGTGAGACCTGTG	RT-qPCR
mKap1 ex4 R	CTGTTCGCTCTCCATCTCGAG	RT-qPCR
mRhox6 ex2 F	CTCAACTGCGGTACAGACG	RT-qPCR
mRhox6 ex2-3 R	CGTGCAAGATCCCTCCTTG	RT-qPCR
mUbe2l6 ex3 F	CACCAAGATCTACCACCCCA	RT-qPCR
mUbe2l6 ex3-4 R	GGCCTCCAAGACTTGATAAGG	RT-qPCR
mTlr2 F	CTGAAGAGGACTGTTATGGCC	RT-qPCR
mTlr2 R	ACCGAAACCTCAGACAAAGC	RT-qPCR
mNaprt1_ex4 F	GCAGGGCCGGATAAGAAACT	RT-qPCR
mNaprt1_ex4-5 R	GCCGCAAACCCATCTCTAGT	RT-qPCR
mRbm44_ex1-2 F	ACAGACGACACTTACGAGCA	RT-qPCR
mRbm44_ex2 R	AGTGTCCTCCATTTTTGGGGT	RT-qPCR
mTspan_ex1 F	CCTGGGGAATCGGGAGCC	RT-qPCR
mTspan_ex1-2 R	CGGCTAGCCAGAACAGCA	RT-qPCR
mBrdt_ex15-16 F	ACTCAGCCTCTTTCAGGTGAT	RT-qPCR
mBrdt_ex16 R	GAGCATGGCATTCTGGCTCC	RT-qPCR
mSerpina3m_ex5 F	AAACAAGCTGACCTATCTGGG	RT-qPCR
mSerpina3m_ex6 R	CTGCTTCTGTGCCTGTCTC	RT-qPCR
mDaxx ex3-4 F	TGACTATAGGCCAGGCGTTGA	RT-qPCR
mDaxx ex4 R	CTCATCCAGCCGGTTCATGG	RT-qPCR
Nanog ChIP F	CGGTGATACGTTGGCCTTCT	ChIP-qPCR
Nanog ChIP R	TGAAGACACCACTCACTGCC	ChIP-qPCR
Desert ChIP F	GAGCATCCGAGAGCATGATT	ChIP-qPCR
Desert ChIP R	TTACACTGCACTTGGGCATC	ChIP-qPCR
IAP PBS ChIP F	CGTGAGAACGCGTCGAATAA	ChIP-qPCR
IAP PBS ChIP R	TTCTGGTTCTGGAATGAGGG	ChIP-qPCR
MusD PBS ChIP F	TGAGCTTTGATCAGTATGAAATTG	ChIP-qPCR
MusD PBS ChIP R	GTGAACGGTTCGACTGAGAA	ChIP-qPCR
Lonrf2 ChIP F	CACTGCCATGCAACTTAGTAAC	ChIP-qPCR
Lonrf2 ChIP F	AGTTGCATGGCAGTGTACATA	ChIP-qPCR
LINE-1 ChIP F	GATTACCAGATGGCGAAAGG	ChIP-qPCR
LINE-1 ChIP R	AGTGCTGCGTTCTGATGATG	ChIP-qPCR

Supplementary Table 5 | List of shRNAs (in pLKO.1 vector).

shRNA target	Sequence (5' to 3')	Clone ID	Company	TRC Number (if applicable)
Non-targeting control	CCTAAGGTTAAGTCGCCCTCGCTCGAGCG AGGGCGACTTAACCTTAGG		Addgene (#1864)	
Mouse Atrx	CCGGCCTGTCACTTTCACCTCTCAACTCGA GTTGAGAGGTGAAAGTGACAGGTTTTTG	NM_009530.1-7064s1c1	Sigma	TRCN0000081910
Mouse Atrx	CCGGCCACTAACACTCCTGAGGATTCTCGA GAATCCTCAGGAGTGTTAGTGGTTTTTG	NM_009530.1-1713s1c1	Sigma	TRCN0000081911

Supplementary Table 6 | List of siRNAs.

siRNA description	Sequences (5' to 3')	Cat. No.	Company
siGENOME Non-Targeting siRNA #1	UAGCGACUAAACACAUCAA	D-001210-01-20	Dharmacon
siGENOME Mouse Trim28 (21849) siRNA - SMARTpool	CUAAGAAGCUGAUCUAUUU	D-040800-01	Dharmacon
	GGACAAACAUGCCACACUU	D-040800-02	Dharmacon
	CAAGAUGCUGAAGCCUUU	D-040800-03	Dharmacon
	GAACCAACGUAAACUCUUG	D-040800-04	Dharmacon
siGENOME Mouse Daxx (13163) siRNA - SMARTpool	GCUACAAGUUGGAUAAUGA	D-044803-01	Dharmacon
	CCGCUAAGAUCUAUGUGUA	D-044803-02	Dharmacon
	GACCCAGACUCCUCGUAUU	D-044803-03	Dharmacon
	UCAAUGGGCGUGUCUCUUC	D-044803-04	Dharmacon
siGENOME Mouse H3f3a (15078) siRNA - SMARTpool	ACGCGGAGAACGUGCUUAA	D-042679-01	Dharmacon
	GCCAAACGUGUAACAAUUA	D-042679-02	Dharmacon
	GUAAAGCACCCAGGAAACA	D-042679-03	Dharmacon
	GUGAAGAAACCUCAUCGUU	D-042679-04	Dharmacon
siGENOME Mouse H3f3b (15081) siRNA -SMARTpool	UGAGAGAGAUCCGUCGUUA	D-042835-01	Dharmacon
	CCAGUUGGCUCGCCGGAUA	D-042835-02	Dharmacon
	GAACCAAGCAGACCGCUAG	D-042835-03	Dharmacon
	CACCAAGGCGGCUCGGAAA	D-042835-04	Dharmacon
